# An ORMOSIL-Containing Orthodontic Acrylic Resin with Concomitant Improvements in Antimicrobial and Fracture Toughness Properties

**DOI:** 10.1371/journal.pone.0042355

**Published:** 2012-08-01

**Authors:** Shi-qiang Gong, Jeevani Epasinghe, Frederick A. Rueggeberg, Li-na Niu, Donald Mettenberg, Cynthia K. Y. Yiu, John D. Blizzard, Christine D. Wu, Jing Mao, Connie L. Drisko, David H. Pashley, Franklin R. Tay

**Affiliations:** 1 Department of Stomatology, Tongji Hospital, Huazhong University of Science and Technology, Wuhan, China; 2 Pediatric Dentistry and Orthodontics, The University of Hong Kong, Hong Kong SAR, China; 3 College of Dental Medicine, Georgia Health Sciences University, Augusta, Georgia, United States of America; 4 School of Stomatology, Fourth Military Medical University, Xi’an, China; 5 Quadsil Inc., Midland, Michigan, United States of America; 6 College of Dentistry, University of Illinois at Chicago, Illinois, United States of America; University of Iowa Carver College of Medicine, United States of America

## Abstract

Global increase in patients seeking orthodontic treatment creates a demand for the use of acrylic resins in removable appliances and retainers. Orthodontic removable appliance wearers have a higher risk of oral infections that are caused by the formation of bacterial and fungal biofilms on the appliance surface. Here, we present the synthetic route for an antibacterial and antifungal organically-modified silicate (ORMOSIL) that has multiple methacryloloxy functionalities attached to a siloxane backbone (quaternary ammonium methacryloxy silicate, or QAMS). By dissolving the water-insoluble, rubbery ORMOSIL in methyl methacrylate, QAMS may be copolymerized with polymethyl methacrylate, and covalently incorporated in the pressure-processed acrylic resin. The latter demonstrated a predominantly contact-killing effect on *Streptococcus mutans* ATCC 36558 and *Actinomyces naselundii* ATCC 12104 biofilms, while inhibiting adhesion of *Candida albicans* ATCC 90028 on the acrylic surface. Apart from its favorable antimicrobial activities, QAMS-containing acrylic resins exhibited decreased water wettability and improved toughness, without adversely affecting the flexural strength and modulus, water sorption and solubility, when compared with QAMS-free acrylic resin. The covalently bound, antimicrobial orthodontic acrylic resin with improved toughness represents advancement over other experimental antimicrobial acrylic resin formulations, in its potential to simultaneously prevent oral infections during appliance wear, and improve the fracture resistance of those appliances.

## Introduction

The demand for orthodontic services is increasing and about 60% of the US population requires some form of orthodontic treatment [1]. Thus, there is an increasing need for the use of orthodontic acrylic resins in removable appliances and retainers. The oral cavity harbors a variety of microorganisms, most of which are considered as opportunistic pathogens. Uncontrolled accumulation of bacterial and fungal biofilms on or surrounding dental devices may contribute to dental caries [2], periodontal disease [3], and chronic atrophic candidiasis [4]. This is particularly important for removable acrylic resin appliances, which have to be worn for a prolonged period of time, and are often exposed to intraoral conditions with low salivary pH levels. Removable orthodontic appliances often promote the accumulation and colonization of dental plaque bacteria including *Streptococcus mutans* [5] and *Candida albicans* [6]. Growth of biofilms on the surface of these appliances may compromise the oral health of patients, and jeopardize the efficiency of orthodontic treatment. Porosities on the outer and inner surfaces of orthodontic acrylic appliances also create favorable conditions for microbial colonization [7].

Mechanical cleaning of acrylic appliances is helpful in reducing biofilm or its formation, particularly with the adjunctive use of antimicrobial solutions containing chlorhexidine, cetylpyridinium [8] or NitrAdine [9]. However, such measures rely primarily on a patient’s compliance, and may be compromised in pediatric, geriatric, and handicapped individuals. Hence, direct incorporation of antimicrobials into orthodontic acrylic resin has been proposed, for sustained release of the antimicrobial component into the oral cavity. Silver nanoparticles, either as-prepared [10]–[13], or generated *in-situ* during resin polymerization [14], have been impregnated in acrylic resins to confer antimicrobial activity to the removal appliances. Such antibacterial effects are mainly attributed to the release of silver nanoparticles or ions upon immersion of the resin in water. As acrylic resin is a rather hydrophobic polymer with low water uptake, releasing of silver nanoparticles from the bulk resin is limited [12], [13]. Using a hybrid process of plasma-based ion implantation and deposition, Shinonaga and Arita introduced antibacterial properties to acrylic resins by incorporating fluorine and silver ions [15]. Leaching of antimicrobials from a composite often displays a burst-release phase during the first few weeks after application. This phase is followed by a much lower, tail-release that is too low to be effective. This reduction is unacceptable for orthodontic appliances, which are intended to remain in the oral cavity for months to years, wherein the antibacterial agent may be depleted. Moreover, elution of antimicrobial agents may result in deterioration of the mechanical properties of the acrylic resin over time. This reduction renders the appliance more susceptible to fracture, due to its low resistance to impact, low flexural strength, or low fatigue strength [16].

Recently, a new class of methacrylate macromonomers - quaternary ammonium methacryloxy silicate (QAMS), has been introduced [17]. By using tetraethoxysilane (TEOS) as the anchoring unit, one molecule of 3-(trimethoxysilyl)propyldimethyloctadecyl ammonium chloride (SiQAC) and three molecules of 3-methacryloxypropyltrimethoxysilane (3-MPTS) are attached via a silane-based, sol-gel route. Due to the antimicrobial activity endowed by the long, lipophilic –C_18_H_37_ alkyl chain of SiQAC [18], this process results in an organically modified silicate (ORMOSIL)-based antimicrobial, which can copolymerize with methacrylate monomers. Incorporating QAMS into the acrylic polymer matrix appears to be a promising alternative for formulating an antimicrobial orthodontic acrylic resin that kills oral microorganisms upon contact [19]. Because the cross-linked backbone of a polymer determines its mechanical behavior, polymers prepared from SiQAC-functionalized macromonomers may have improved toughness and damping properties, due to the flexibility of the siloxane backbone, as compared with rigid C–C bonds [20].

The present study examined the antibacterial and antifungal activities, wettability, flexural properties, fracture toughness, and water sorption and solubility of a commercially available, pressure-processed, orthodontic acrylic resin that had been incorporated with different concentrations of QAMS. The hypotheses tested were (1) QAMS-containing orthodontic acrylic resins exhibit antibacterial and antifungal properties, and (2) incorporating QAMS into orthodontic acrylic resins results in improved fracture toughness properties without adversely affecting other physical and mechanical properties.

## Materials and Methods

### Preparation of QAMS

The QAMS, being an organically modified silicate (ORMOSIL), was synthesized by sol-gel reaction in the manner described previously [17]. Briefly, tetraethoxysilane (TEOS) and 3-methacryloxypropyltrimethoxysilane (3-MPTS) were obtained from Sigma-Aldrich (St. Louis, MN, USA). AEM 5772 antimicrobial agent (72% SiQAC in methanol) was obtained from W.M. Barr & Company (Memphis, TN, USA). The QAMS monomer mixture was prepared by adding TEOS, SiQAC, and 3-MPTS in a 1∶1∶3 molar ratio. Stoichiometric molar concentrations of acidified MilliQ water (16 moles; pH 2.5) was added to the QAMS monomer mix to completely hydrolyze the organosilanes. Subsequent condensation of the silanol groups was conducted at pH 7.4 by adding sufficient 1 M NaOH to the previously hydrolyzed QAMS. The precipitate was vacuum-stripped to remove the by-products of hydrolysis and condensation from the condensate, producing a rubbery ORMOSIL with a final yield of 57.9 wt%. The water-and-alcohol-depleted QAMS was characterized using ^29^Si cross polarization-magic angle spinning nuclear magnetic resonance spectroscopy (CP-MAS NMR) and attenuated total reflection-Fourier transform infrared spectroscopy (ATR-FTIR).

### Preparation of QAMS-containing Orthodontic Acrylic Resin

A commercially available, auto-polymerizing orthodontic acrylic resin system (Ortho-Jet; Lang Dental Manufacturing Co. Inc., Wheeling, IL, USA) was used. The QAMS condensate, being insoluble in water, was serendipitously discovered to be soluble in methyl methacrylate (MMA). This finding enabled us to create different concentrations of the water-and-alcohol-depleted QAMS condensate in the Ortho-Jet MMA liquid component (Lot 144211DM/01AB, Ref #1304, clear, Ortho-Jet Liquid, Lang Dental), resulting in five QAMS-MMA comonomer resin blends: 0 (control), 1, 5, 10, and 15% by weight of QAMS, respectively. Each QAMS-MMA comonomer was mixed with the powder component (Lot 602711AR/01AM, Ref #1330, Lang Dental) (consisting of polymethyl methacrylate pre-polymer and diethyl phthalate) in a liquid:powder mass ratio of 2∶3. The dough was packed into different molds as specified by the International Organization for Standardization (ISO 20795-2) [21], and processed in a pressure curing unit (Aquapres™, Lang Dental) according to the manufacturer’s instruction. The polymerized orthodontic acrylic resin specimens contained 0 (control), 0.4, 2, 4, and 6% by weight of QAMS, respectively, and were characterized using ATR-FTIR.

### Solid-state ^29^Si CP-MAS NMR

Solid-state CP-MAS silicon NMR was performed at ambient temperature using a 270 MHz spectrometer (JEOL, Tokyo, Japan) equipped with a 7 mm Magic Angle Spinning (MAS) probe. Spectra were acquired in the ^1^H→^29^Si cross polarization (CP) mode, using a MAS frequency of 4 kHz, with a 45 degree pulse angle of 5 µsec. The ^1^H Larmor frequency for ^29^S was 53.76 MHz. Chemical shifts were referenced to external tetramethylsilane (TMS) at 0 ppm.

### ATR-FTIR

Infrared spectra were recorded between 4,000-400 cm^−1^ using a Fourier-transform infrared spectrometer (Nicolet 6700, Thermo Scientific, Waltham, MA, USA) with an attenuated total reflection (ATR) at a resolution of 4 cm^−1^ and averaging 32 scans per spectrum. Spectra of the QAMS condensate free of water and alcohol, the MMA monomer component, and the polymerized orthodontic acrylic from the five groups were collected.

### Biofilm Preparation and Antimicrobial Efficacy Evaluation

#### Preparation of bacterial suspensions

*Streptococcus mutans* ATCC 36558 (ATCC, Manassas, VA, USA) and *Actinomyces naeslundii* ATCC 12104 were cultured in Brain Heart Infusion (BHI) broth (Difco, Becton-Dickinson and Co., Sparks, MD, USA) supplemented with 50 mM sucrose (pH 7.2). *Candida albicans* ATCC 90028 was cultured in Yeast Nitrogen Base (YNB; Difco) supplemented with 50 mM glucose (pH 7.2). Cells were harvested from 24-hour fresh culture by centrifugation at 4000 rpm for 10 min. The respective cell pellet was washed three times with sterile phosphate buffered saline (PBS, 0.01 M, pH 7.2), re-suspended in 100 mL of the respective growth medium, and adjusted to a concentration of 10^7^ CFU/mL before use.

#### Growth of biofilm inside an oral biofilm reactor

Disks (6 mm diameter×1 mm thick; N = 6/group) from the control group and the four QMAS-containing experimental groups were prepared to fit the specimen holder of an oral biofilm reactor. All disks were disinfected under ultraviolet light for 2 h prior to experiments. Each microbe was used individually for the formation of single-species biofilms. The oral biofilm reactor consisted of a reactor vessel and a specimen holder (Figure S1) containing 18 recessed holders for insertion of substrate disks over which microbial biofilms could be grown [22]. The six acrylic disks from each group were affixed to the sample ports of the specimen holder, and incubated in pooled sterile saliva for 1 h at 37°C, to create a salivary pellicle on the surface. The sterile pooled saliva was obtained by collecting whole saliva from three healthy volunteers without stimulation (flow rate 0.25 mL/min; pH 7.3). The pooled saliva was centrifuged at 2000 rpm for 15 min to remove cellular debris, oral microorganisms and particles, and the supernatant was collected and mixed with dithiothreitol (Sigma-Aldrich, 2.5 mmol/L) to reduce salivary protein aggregation. The mixture was centrifuged again at 2000 rpm for 15 min at 4°C. The supernatant was collected and filter-sterilized through disposable, sterile 0.22 µm filters (Nalge Numc International, Rochester, NY), and stored at −20°C until use.

The specimen holder with the acrylic disks was then transferred to the reactor vessel which was subsequently filled with *S. mutans, A. naeslundii* or *C. albicans* cell suspension (10^7^ CFU/mL). The assembly was first incubated for 90 min at 37°C, in an orbital shaker incubator at 50 rpm, to develop the adhesion phase of the respective biofilm. Following the adhesion phase, the specimen holder was removed, rinsed carefully with 100 mL of PBS (0.01 M, pH 7.2), and transferred aseptically to a new, sterile reactor vessel, which held the disks on a fixed stage in 200 mL of the respective nutrient medium. The assembly was placed over an orbital incubator (37°C; 50 rpm), and connected to several vessels (nutrient, sucrose solution, and waste) and to an infusion pump, to complete the *in vitro* artificial mouth system. A desired flow rate was obtained before allowing biofilm formation to proceed for 48 h. The flow rate was adjusted according to the chemostat mode at a dilution rate of 0.10 h^−1^. *S. mutans* and *A. naeslundii* biofilms were grown under anaerobic conditions (5% carbon dioxide, 10% hydrogen and 85% nitrogen). *C. albicans* biofilms were grown under aerobic condition. At the end of the 48-h growth period, the specimen holder with the acrylic discs was aseptically removed, and immersed in 100 mL of sterile PBS to remove non-adherent cells. The acrylic disks with adherent biofilms were then retrieved for further investigation.

#### Confocal laser scanning microscopy (CLSM)

Microbial viability within the biofilms was assayed with a staining kit (LIVE/DEAD *Bac*Light, Molecular Probes, Eugene, OR, USA). The latter contains the fluorescent stain SYTO-9 to identify live microbes, and propidium iodide (PI) for visualization of dead microbes. Stock solutions of SYTO-9 and PI were diluted 2000-fold, and staining of the biofilms present on the surface of the acrylic disks was allowed to proceed for 15 min. After incubation with the dyes, the biofilm-containing acrylic discs were placed on glass slides and imaged using a CLSM (Fluoview FV1000, Olympus, Tokyo, Japan) at 40 X magnification, using excitation wavelengths of 488 nm and 568 nm for SYTO-9 and PI, respectively. For each acrylic disk, an image stack (Z-stack) was obtained at a location that was characteristic of that biofilm. Images were acquired at a Z-step of 2 µm (i.e. distance between each image in a stack), beginning from the bottom of the biofilm that was in contact with the acrylic surface, to the top of the biofilm. Each image had a field size of 318 µm×318 µm.

#### Analysis of biofilms

Images from each Z-stack were analyzed using software (bioImageL v.2.1, Faculty of Odontology, Malmö University, Malmö, Sweden) [23]. Three-dimensional image analysis was performed for the *S. mutans* and *A. naeslundii* biofilms. For each biofilm, the biovolume within the first 24 µm of each Z-stack (i.e. 1^st^ –13^th^ images) was analyzed for the percentage distribution of live and dead microbes. The first image of each Z-stack, representing direct contact of the microbes with the pellicle on the acrylic disk surface, was also analyzed, to identify the effect of contact-killing by the immobilized antimicrobial agent. Data obtained from the biovolumes of interest (first parameter), and from the basal layer of the biofilms (second parameter) in the 5 groups were analyzed separately using one-way ANOVA and post-hoc Tukey multiple comparison tests, after validating the normality and homoscedasticity assumptions of the data set. If either assumption was violated, the data set was transformed logarithmically prior to the application of the aforementioned parametric statistical analysis methods. For all analyses, statistical significance was preset at á = 0.05.

As *C. albicans* only formed biofilms on non-QAMS-containing acrylic surfaces, and attached poorly as a discontinuous monolayer to QAMS-containing acrylic surfaces, 2-D image analysis was adopted for evaluation. Briefly, a merged image obtained from a representative location of each acrylic disk was used for 2-D analysis of: a) the percentage distribution of total biomass (i.e. both live and dead fungi) within the imaged field, and b) the percentage distribution of green biomass (i.e. live fungi) within the total biomass. The data set obtained for each of these parameters was analyzed using the aforementioned statistical methods, under the same statistical assumptions.

### Remnant and Leachable Quaternary Ammonium Species

The number of QAMS species exposed on the acrylic specimen surface after water-aging was assessed using the fluorescein staining method [19], [24]–[25]. This method was based on the ability of a single fluorescein molecule to bind to the N^+^ charge of a quaternary ammonium molecule. Briefly, acrylic disks (14.5 mm diameter × 0.4 mm thick; 75 mg ±3 mg) containing 0, 0.4, 2, 4 and 6 wt% QAMS were prepared (N = 8). Each disk was aged in 2 mL of deionized water in a container at 37°C for 7 days under continuously shaking. After aging, each disk was retrieved from the aging solution, and placed into an individual vial containing 2 mL of a 10 mg/mL stock solution of fluorescein sodium salt (Sigma-Aldrich). Specimens were allowed to stand in the dark at room temperature for 10 min. The specimens were then removed and placed into another container into which 20 mL of distilled water was placed and shaken by hand vigorously. This process was continued for five times, to ensure that all unbound fluorescein had been removed. Extraction of bound fluorescein from the dried QAMS-containing acrylic resin disks was performed in the dark, by ultrasonicating each specimen in a vial containing 1.8 mL of a 0.1 wt% cetyltrimethylammonium chloride stock solution (Sigma-Aldrich). Ultrasonication was performed for 20 min to allow desorption of the bound dye from the acrylic disc. Release of the captured fluorescein from the extracted solution was achieved by adding 0.2 mL of 100 mM of phosphate-buffered saline (pH 8.0) to each vial. Two milliliters of this solution mixture was then placed into a test cuvette of a UV-VIS spectrophotometer (Chem2000, Ocean Optics, Dunedin, FL) to obtain an absorbance spectrum of the bound fluorescein. The absorbance of the solution mixture was measured at 501 nm. The extinction coefficient of fluorescein in the solution was taken to be 77 mM^−1^cm^−1^ [19]. The number of nanomoles of fluorescein in the cuvette was calculated from a calibration graph, relating fluorescein concentration and absorbance, and then divided by the surface area of the specimen to arrive at an N^+^ charge density (nM/cm^2^).

As the fluorescein staining test did not account for the amount of QAMS released into solution as an unbound component, the amount of leached QAMS into the aged solution used for storing the QAMS-containing acrylic was determined using the bromophenol blue assay [24], [26]. For this assay, acrylic resin disks prepared by incorporating 5 wt% SIQAC, which does not contain an methacryloxy group and will leach readily from the processed acrylic, were used as the positive control and aged similar in deionized water for 7 days. Bromophenol blue is a dye which forms a complex with quaternary ammonium compounds and results in a shift of lmax from 593 nm to 603 nm as a result of complex formation. Briefly, an aqueous solution of 0.001 wt% bromophenol blue was prepared in deionized water and this solution was buffered with sodium carbonate solution to pH 7.0 to avoid absorption changes owing to pH fluctuations. A 0.5 mL of the aged solution from each sample was collected at 24 h, mixed with 1.5 mL bromophenol blue solution and agitated gently for 10 min. Absorbance of the leachate was recorded using the UV-Vis spectrophotometer at 603 nm. The absorption values of different concentrations of SiQAC in deionized water (from 0 to 1.451 mM) were recorded to establish a standard curve to obtain the molar concentration of N^+^ in the respective aged solutions. The cumulative concentration of quaternary ammonium species from the leachate was determined from the calibration curve (N = 8). The data obtained from the SIQAC-containing control acrylic and the 5 QAMS-containing acrylic were analyzed with Kruskal-Wallis ANOVA and Dunn’s multiple comparison tests at α = 0.05.

### Contact Angle Measurement

Polymerized acrylic disks (N = 10) from the five groups were prepared as previously described. The disks were immersed in methanol for 24 hours to remove the unreacted monomer and then vacuum-dried. A commercial contact angle measuring instrument (EasyDrop DSA-20, Krüss, Hamburg, Germany) was used to determine static contact angles of MilliQ water (5 µL) dispensed over each acrylic resin disk surface at 23°C and 50% relative humidity. That volume was computer controlled using a stepper-motor-controlled actuator on a microsyringe. Digital video files were recorded of the silhouette of the surface profile of each water droplet at the rate of 1 image/sec for 60 sec. Static contact angles at each time interval were calculated using the software supplied with the device (Drop Shape Analysis V 1.92, Krüss). For each acrylic resin disk, the static contact angle obtained 30 sec after deposition was taken as the equilibrium angle value. The relation between QAMS concentration in the orthodontic acrylic and the mean equilibrium contact angle was investigated using regression analysis, and the correlation between these two parameters were analyzed using the Pearson Product Moment statistic at á = 0.05. In addition, the correlation between the mean equilibrium contact angle and the extent of *C. albicans* adhesion to the 5 groups of orthodontic acrylic was analyzed using the same statistical test and limits.

### Flexural Properties

The ultimate flexural strength and flexural modulus of the QAMS-containing orthodontic acrylic specimens were evaluated according to the specifications outlined in ISO 20795-2 [21]. Accordingly, two acrylic plates (65×40×5 mm^3^) were fabricated for each group. Each plate was sectioned lengthwise into three strips using a precision cutting machine (Isomet 500, Buehler, Lake Bluff, IL, USA) under water cooling to prevent overheating. The strips were then ground to the required length, width, and height of 64 mm×10.0×3.3 mm, with an automatic grinding and polishing unit (Ecomet III, Buehler) under water cooling, using metallographic grinding paper (Leco, St. Joseph, MI, USA) with a grain size of 15 µm. Specimens free of porosity were selected for flexural testing (N = 10). The resulting bar width and height were recorded, and the specimens were stored in deionized water at 37±1°C for 50±2 hours, prior to flexural testing. The specimens were retrieved from water storage, and placed on the supports of a three-point bending fixture that was immersed in a 37°C temperature-controlled water bath. The distance between the supports was 50 mm. An increased loading force was applied through a universal testing machine (Model 5844 Instron Corp., Norwood, MA, USA), at a constant displacement rate of 5±1 mm/min, until the specimen fractured.

The flexural strength (σ, in MPa) was calculated from the equation:


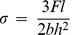
(1)

where *F* is maximum load (N) exerted on the specimen, *l* is the distance (mm) between the supports, *b* is the specimen width (mm) and *h* the specimen height (mm) prior to water storage.

The flexural modulus (*E*, in MPa) was calculated from the equation:


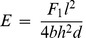
(2)

where *F*_1_ is the load (N) at a point in the straight line portion of the load/deflection curve, *d* is the deflection (mm) at load *F*_1_.

For each parameter (ultimate flexural strength or flexural modulus), the data were compared with the ISO requirements. Each data set was further analyzed using a one-way ANOVA and the Tukey multiple comparison test at α = 0.05, using the statistical assumptions described previously.

### Fracture Toughness: Maximum Stress Intensity Factor

Following the specifications outlined for orthodontic base polymers (ISO 20795-2) [21], two acrylic specimen plates were sectioned breadth-wise, and ground under wet conditions to produce specimens (N = 10) with length (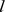), width (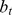), and height (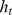) of 39 mm×4.0 mm (±0.2 mm)×8.0 mm (±0.2 mm), respectively, using the same equipment as in flexural strength testing. A pre-crack with depth of 3.0±0.2 mm was cut along the centerline of each specimen, followed by the introduction of a 100–400 µm sharp notch on the bottom of pre-crack. Specimen width and height were recorded. The specimens were stored in deionized water at a temperature of 37°C for 7 days, and then conditioned in deionized water at 23°C for 1 h prior to testing. After conditioning, the specimen strip was blotted dry and placed on the supports of a three-point bending fixture with the notch facing exactly opposite the load plunger. The distance between the supports was 32 mm. A loading force was applied to the specimen at constant displacement rate of 1.0 mm/min until maximum load was achieved. The test was considered finished when the applied load was reduced to 5% of the maximum value, or less than 1.0±0.2 N. The entire load/deflection curve was recorded and, after completing the tests, the total crack length (consisting of the length of the pre-crack (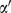 in mm) and the crack length (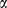 in mm) was determined based on three measurements of the distance between the specimen surface and the area fractured in the test.

The stress intensity factor describes the stress necessary for propagating a pre-existing flaw in a material, and is commonly used to measure the fracture toughness of many materials. The higher the value, the more resistant to crack propagation and fracture is a specimen (i.e. the tougher is the material). The maximum stress intensity factor (K_max_, in MPa·m^1/2^) was calculated from the equation:


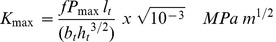
(3)

f is a geometrical function dependent on *x*:


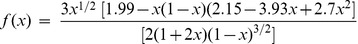
(4)

and


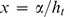
(5)

where Pmax is the maximum load (N) exerted on the specimen, 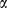 is the total crack length, 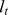 is the distance (mm) between the supports, 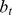 is the width (mm), and 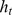
the height (mm) of the specimen measured prior to water storage.

### Fracture Toughness: Work of Fracture

The work of fracture describes the energy required to form two new, separated interfaces along the crack line. The total fracture work (W_f_, in J/m^2^) was calculated from the equation:


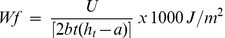
(6)

where U is the recorded area under the load/deflection curve (N·mm).

For each parameter (K_max_ or W_f_), the data were compared with the ISO requirements. Each data set was further analyzed using one-way ANOVA and Tukey multiple comparison test at α = 0.05, using the statistical assumptions described in Section 2.4.4 (Note: the K_max_ data set was first transformed into the square of the original values prior to analysis, to satisfy those assumptions). In addition, the relation between QAMS concentration in the orthodontic acrylic and the mean K_max_ or W_f_ was investigated using regression analysis. The correlations between QAMS concentration and mean K_max_, and between QAMS concentration and mean W_f_, were further analyzed separately using the Pearson Product Moment statistic at á = 0.05.

### Water Sorption and Solubility

Water sorption and solubility were determined according to the ISO specification for orthodontic base polymers (ISO 20795-2) [21], except for specimen dimension (50±0.1 mm diameter, 0.4±0.05 mm thick). In addition, diffusion coefficients for the 5 groups of orthodontic acrylic were also compiled to compare their kinetic water sorption over immersion time.

After polymerization, specimens (N = 10) were stored at 37°C in a desiccator containing silica gel. The specimens were repeatedly weighed in an electronic analytical balance after 24 h until a constant mass (m_1_) was obtained (i.e. the difference between two successive readings was less than 0.2 mg). At this point, the diameter and the thickness of the specimens were measured for determining the volume (V) of each specimen. The specimens were then immersed in water at 37°C for 7 days. To obtain the data necessary to compute diffusion coefficients during water sorption, specimens were weighed every 1.5 hours for 9 hours during the first day, every 12 hours during the second day and every 24 hours until the end of the 7-day period. The final weighting on the 7th day of water immersion was recorded as m_2_. The specimens were reconditioned to a dry, constant mass (m_3_) following the same protocol as for conditioning of the specimens prior to water immersion. Water sorption (W_sp_, µg/mm^3^) and water solubility (W_sl_, µg/mm^3^) were calculated using the following equations:


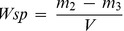
(7)


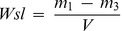
(8)

Diffusion coefficients were determined using the conventional solution to Fick’s 2^nd^ law of diffusion for a plane sheet, by plotting the 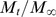 ratios as a function of the square root of time. This calculation was based on the assumption that the diffusion process is linear during the early stage of water sorption (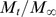
_∞_≤0.5) [27]. The diffusion coefficients of water (D; cm^2^/s) for the 5 orthodontic acrylic groups were calculated using the approximation [28]:


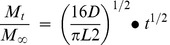
(9)

where 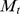 is the mass gain (mg) at time t, 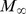
is the mass gain (mg) at equilibrium and L is the thickness (cm) of the specimen.

The water sorption and water solubility data were compared with the corresponding ISO requirements. These data, and those of the diffusion coefficient were analyzed separately using one-way ANOVA and Tukey multiple comparison test at á = 0.05, in accordance with the statistical assumptions described previously.

### Cytotoxicity of QAMS-containing Orthodontic Acrylic Resin

The cytotoxicity of QAMS-containing acrylic was investigated using a murine dental papilla-derived odontoblast-like cell line (MDPC-23). The MTT ((3-(4,5-Dimethylthiazol-2-yl)-2,5-diphenyltetrazolium bromide) assay was used to examine the effects of QAMS-containing acrylic on mitochondrial metabolic activity. Flow cytometry was used to identify the effects of QAMS-containing acrylic on cell death–induced plasma membrane permeability to fluorescent dyes and DNA stains. As remnant MMA within the polymerized PMMA acrylic is cytotoxic [29], experiments were performed before and after the processed QAMS-containing acrylic were subjected to methanol extraction of the residual MMA. Details of the experimental protocols are presented in Supporting Information (Text S1).

## Results

### Compositional Analysis

Solid-state ^29^Si CP-MAS NMR of the water-and-alcohol-depleted QAMS condensate revealed two broad resonance peaks at −68.37 ppm that are assigned to silicon in the RSi(OSi)_3_ species (T^3^ bonding), and at −58.41 ppm that is assigned to silicon of the RSi(OH)(OSi)_2_ species (T^2^ bonding) (Figure S2). The presence of T^3^ and T^2^ functionalities confirms the existence of the covalent linkage between the organic alkoxy groups and the silicate backbone. The broad peaks are indicative of the heterogeneous nature of the condensate, and possibly others within the network formation. No Q-series (siloxane, single silanol and germinal silanols) could be identified from the spectra, indicating that the final condensed product is an organically modified silicate (ORMOSIL).

The infrared spectrum of the unpolymerized QAMS condensate from the fingerprint region (900–1800 cm^−1^; see Figure S3 and Text S2) revealed a broad band peaking at 1040 cm^−1^ that is characteristic of the Si-O-Si linkages. In addition, the band at 1637 cm^−1^ is indicative of C = C derived from unpolymerized methacrylate functionalities. The latter was also identified in the Ortho-Jet MMA liquid component, but absent from the powder-only component. This band was almost completely absent after polymerization of the QAMS-free and QAMS-containing acrylic resin. The presence of a small band (1637 cm^−1^) in the acrylic resins containing 4 and 6 wt% QAMS suggests that addition of the QAMS component to this extent reduces overall conversion of the PMMA resin, but could also represent unreacted silane functionalities introduced by the QAMS and not reacted with the PMMA. The effect of QAMS addition to the orthodontic acrylic resin could be identified from the increasing prominence of the Si-O-Si linkages at approximately 1040 cm^−1^.

### Antimicrobial Activity

**Figure 1:**
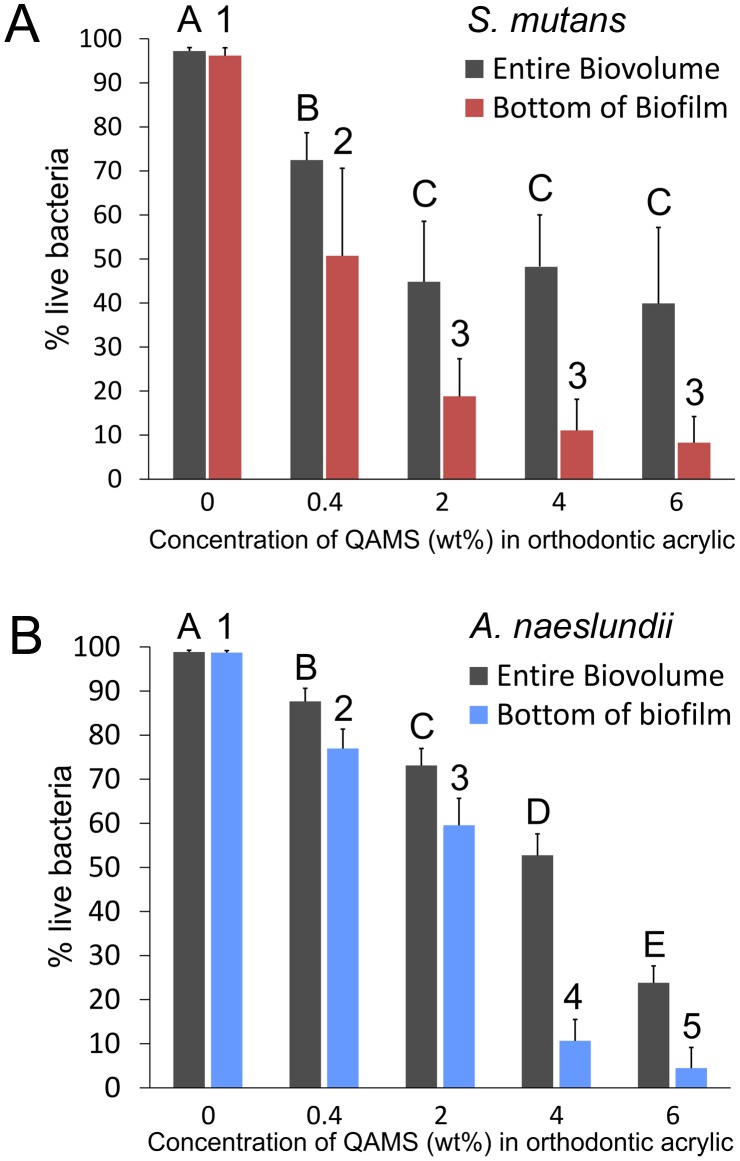
Percentage distribution (mean and standard deviation) of live bacteria within the biovolumes of interest (i.e. the volume of biofilm from the base to the 24^th^ µm of a z-stack), and within the biomass at the basal layer of biofilms that were grown on orthodontic acrylic disks prepared from the control (0 wt% QAMS) and the four experimental groups (0.4–6 wt% QAMS) (N = 6). **A**. *Streptococcus mutans* biofilms. **B**. *Actinomyces naeslundii* biofilms. For each parameter (upper case letters for biovolume and numerals for bottom of biofilm), groups labeled with the same category of descriptors (i.e. upper case letters, lower case letters, numeric) are not statistically significant (P>0.05).

**Figure 2:**
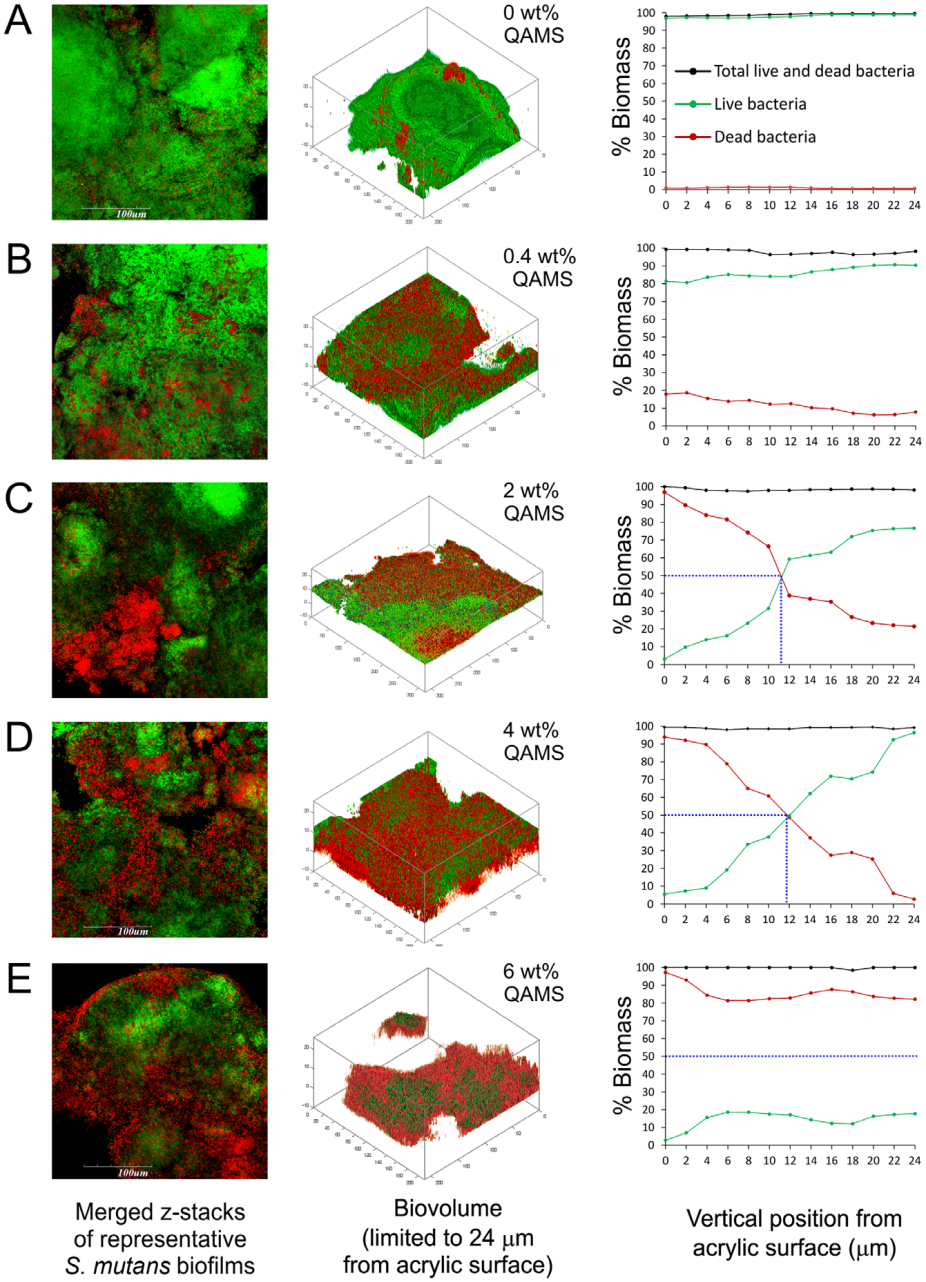
Representative *Bac*Light-stained 48-h *S. mutans* biofilms grown on the surfaces of orthodontic acrylic resins with different QAMS concentrations. **A**. 0 wt%. **B**. 0.4 wt%. **C**. 2 wt%. **D**. 4 wt%. **E**. 6 wt%. Green: live bacteria; Red: dead bacteria. The first column represents merged CLSM images of the biofilms. The second column represents 3-D plots of each biofilm within a biovolume extending from the 1^st^ (basal) to the 13^th^ layer of a Z-stack (i.e. 24 µm thick). The 3rd column represents the percentage distribution of live and dead bacteria within the biomass at each level of the biofilm (Z-step  = 2 µm). For **C**–**E**, the blue dotted line denotes the levels with 50% dead biomass.

**Figure 3:**
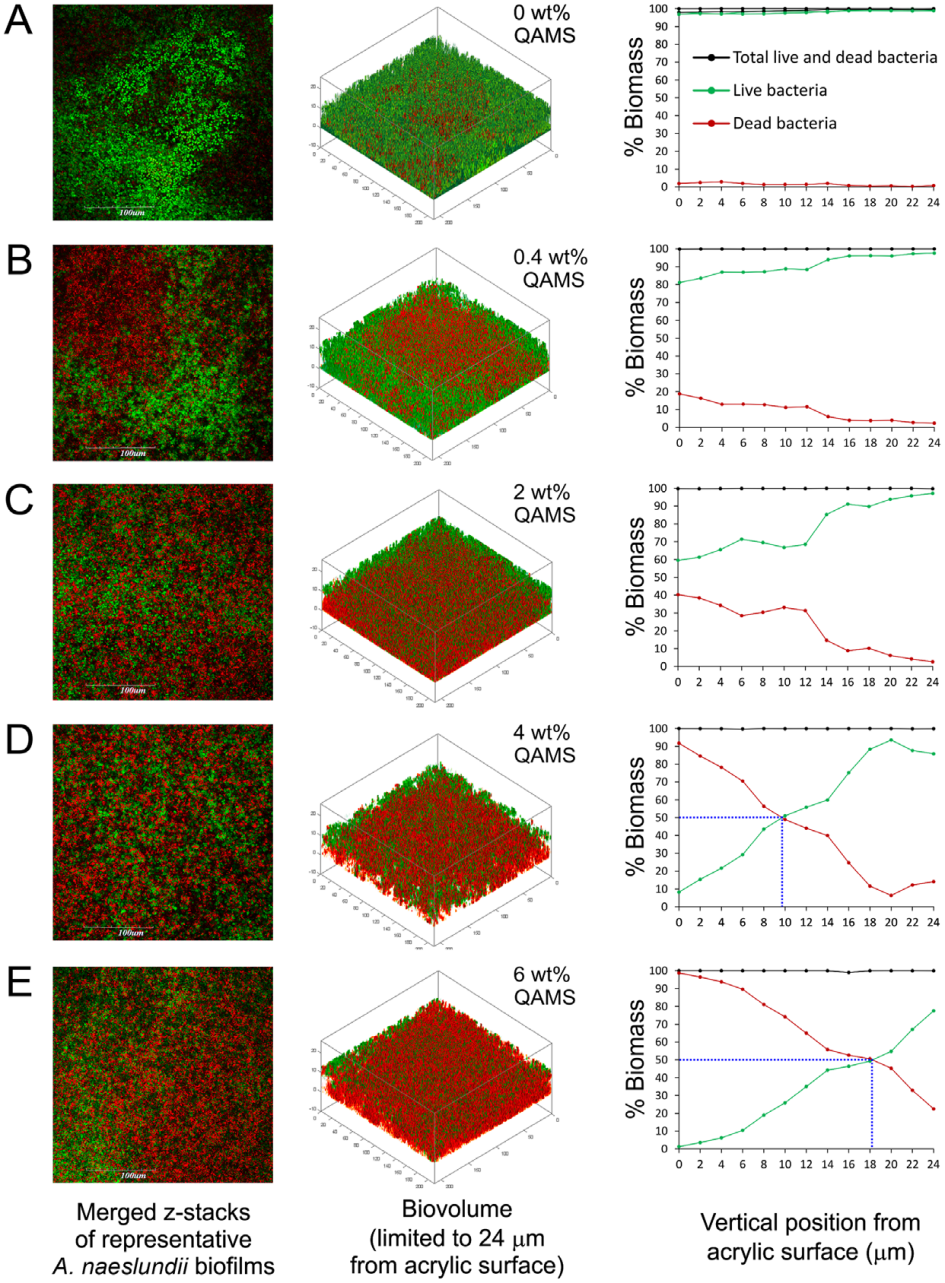
Representative *Bac*Light-stained, 48-h A. naeslundii biofilms grown on the surfaces of orthodontic acrylic resins with different QAMS concentrations. **A**. 0 wt%. **B**. 0.4 wt%. **C**. 2 wt%. **D**. 4 wt%. **E**. 6 wt%. Green: live bacteria; Red: dead bacteria. Column designations are the same as Figure 2. For the scatter plots in the right column of **C**–**E**, the blue dotted line denotes the levels with 50% dead biomass.

The percentage distributions of live bacteria present in *S. mutans* and *A. naeslundii* biofilms are shown in Figures 1A and 1B, respectively. Immobilization of QAMS within the PMMA polymer matrix did not inhibit adhesion of *S. mutans* and *A. naselundii* bacteria species to the pellicle-coated acrylic surfaces (see left columns in Figures 2 and 3). However, there were significant reductions of live bacteria within the biofilms that were dependent on the concentration of QAMS into the acrylic resins. For all QAMS-containing acrylic resins, the percentage of live bacteria at the bottom of the biofilms was lower than those detected within the biovolume of interest (i.e. the volume of biofilm from the base to the 24^th^ µm of a z-stack). For both locations, resins containing 2, 4, and 6 wt% QAMS provided more effective killing than the resin containing 0.4 wt% QAMS, but were not significantly different from one another. Figure 2 (*S. mutans*) and Figure 3 (*A. naselundii*) contain merged images of the architecture of *Bac*Light-stained biofilms grown over the surface of orthodontic acrylic resins containing increasing concentrations of QAMS (left column). The distribution of live (green) and dead (red) bacteria within the biovolumes of those biofilms are depicted as 3-D plots in the center column. The percentage distributions of live and dead bacteria from the biomass at each level are summarized in the line plots in the right column.

**Figure 4:**
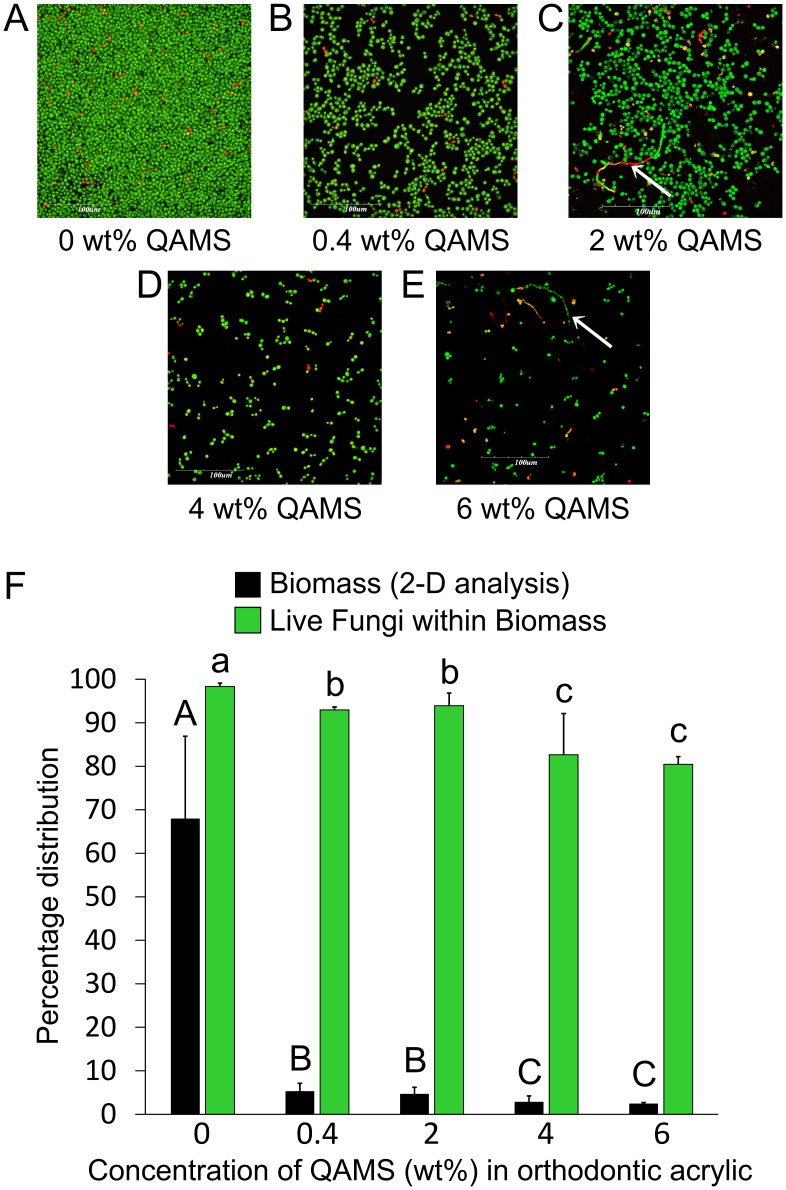
Representative merged images illustrating the adhesion and growth of *C. albicans* on orthodontic acrylic surfaces containing A. 0 wt% QAMS (control). **B**. 0.4 wt% QAMS. **C**. 2 wt% QAMS. **D**. 4 wt% QAMS. **E**. 6 wt% QAMS. **F**. Two-dimensional analysis of the growth of *C. albicans* on the surfaces of orthodontic acrylic resins with different QAMS concentrations (N = 6). *C. albicans* did not form biofilms on acrylic surfaces containing QAMS. Percentage distributions of the fungal biomass with the field of examination are shown as black columns, and the percentage distributions of live fungus within the biomass are depicted as green columns. For each parameter, groups identified with the same category of descriptors (i.e. upper case letters for biomass and lower case letters for live fungi) are not statistically significant (P<0.05). Values represent means and standard deviations.

Unlike *S. mutans* and A. *naeslundii*, *C. albicans* formed biofilms only in the QAMS-free acrylic resin that consisted predominantly of yeast cells (Figure 4A). There was minimal yeast-hyphal transition in all the 48 hour biofilms. In the presence of QAMS, *C. albicans* cells detached easily under the continuous flow condition of the reactor vessel and did not mature into biofilms on the pellicle-coated acrylic surfaces. Only discontinuous monolayers of yeast cells were apparent on acrylic resins containing QAMS (Figures 4B–4E). Two-dimensional image analysis of the area occupied by the total (live and dead) biomass within the field of view, and the percentage distributions of live *C. albicans* within those biomasses are shown in Figure 4F. For both parameters, acrylic resins containing 4 and 6 wt% QAMS significantly inhibited *C. albicans* attachment and viability than those containing 0.4–2 wt% QAMS. All of the acrylic resins containing QAMS promoted significantly less *C. albicans* attachment compared to the QAMS-free acrylic resin.

### Remnant and Leachable Quaternary Ammonium Species

**Figure 5:**
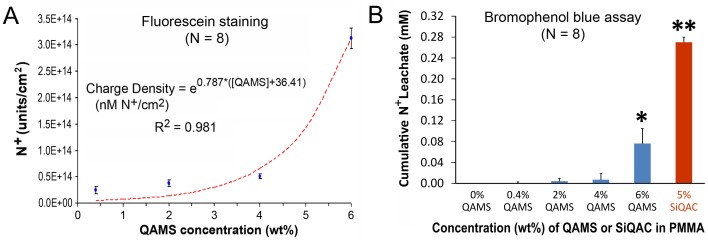
Colorimetric assays for examining the amount of A. Remnant quaternary ammonium charges present on the surface of the acrylic (fluorescein staining) after a 7-day water-aging period, and B. Cumulative amount (in mM) quaternary ammonium moieties present in the leachate after the 7-day water-aging period. Columns indicated by different symbols are significantly different from those without symbols (P<0.05).

Figure 5A presents the plot of mean charge density/unit area of the N^+^ species present on the QAMS-containing acrylic surfaces after a 7-day water aging period. The relation between charge density and QAMS concentration could be modeled by a power regression model (R^2^ = 0.981). Figure 5B shows the cumulative molar concentrations of N^+^ species in the leachate derived from the QAMS-containing acrylic and the SIQAC-containing acrylic (positive control). There was negligible leaching of N^+^ species from acrylic specimens containing less than 4 wt% QAMS. Leaching of N^+^ species from acrylic specimens containing 6 wt% QAMS was significantly higher, while specimens containing 5% SiQAC (without methacryloxy groups that copolymerized with PMMA) leached the most (P<0.05).

### Physical and Mechanical Properties

The mean equilibrium contact angle for the 5 groups of acrylic with 0, 0.4, 2, 4, and 6 wt% QAMS are 72.6±4.3°, 76.2±2.4°, 83.0±1.4°, 85.2±2.7°, and 89.8±2.3°, respectively. Linear regression provided a good fit of the changes in contact angle with increasing QAMS concentration (r^2^ = 0.93). A highly significant, positive correlation of these two parameters was also identified (Pearson correlation coefficient  = 0.954; P = 0.003). Taken together, the data show that the orthodontic acrylic resin becomes increasingly hydrophobic as the concentration of QAMS increases. However, a significant correlation (P = 0.158) was not found between the changes in contact angle and variations in *C. albicans* total biomass in the QAMS-free and QAMS-containing acrylic resins.

**Figure 6:**
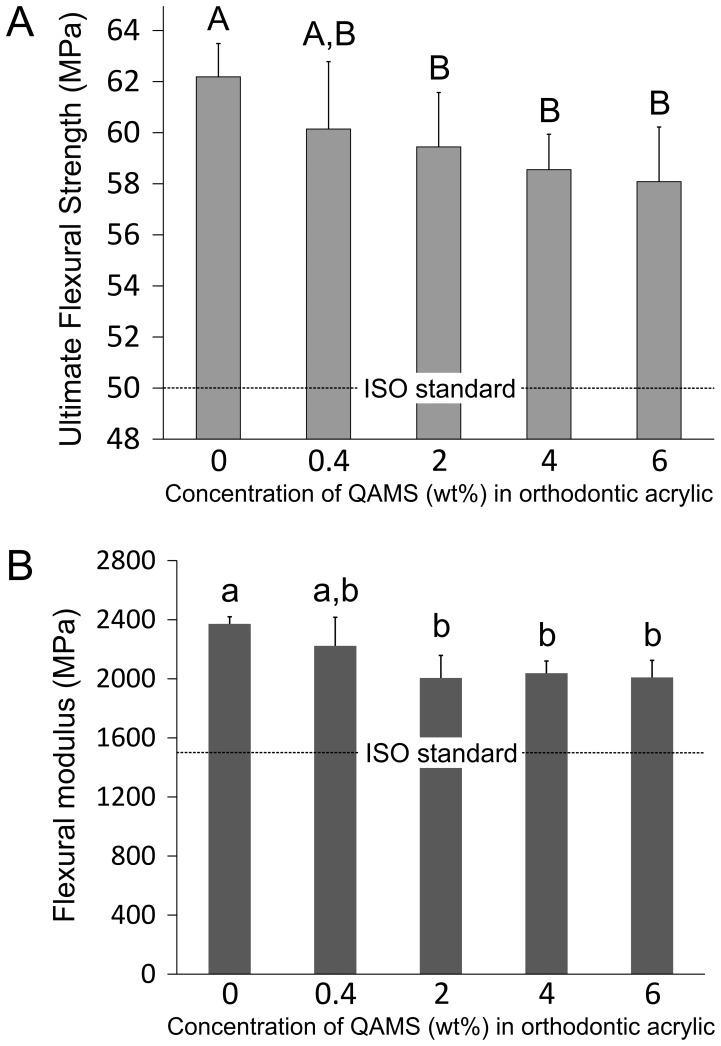
Effects of QAMS concentration on the flexural properties of the orthodontic acrylic resins (N = 10). **A**. Ultimate flexural strength. **B**. Flexural modulus. For each parameter, the ISO requirement is included for comparison (dotted line). For the ultimate flexural strength, groups with the same upper case letter designators are not statistically significant (P>0.05). For flexural modulus, groups with the same lower case letter designators are not statistically significant (P>0.05). Values represent means and standard deviations.

The effects of QAMS incorporation on the flexural properties of orthodontic acrylic resins are shown in Figure 6. A small, but statistically significant decline in ultimate flexural strength (Figure 6A) could be identified after the addition of more than 0.4 wt% QAMS to the orthodontic acrylic resin. Nevertheless, these values are all higher than the ISO requirement for orthodontic base polymers (50 MPa). No additional decline in ultimate flexural strength was observed with further increase in QAMS concentration in the acrylic resin (P>0.05) A similar trend in the reduction of the flexural modulus (Figure 6B) was also seen after QAMS was incorporated into the acrylic resin. Nevertheless, these values were all higher than the ISO requirement for orthodontic base polymers (1500 MPa).

**Figure 7:**
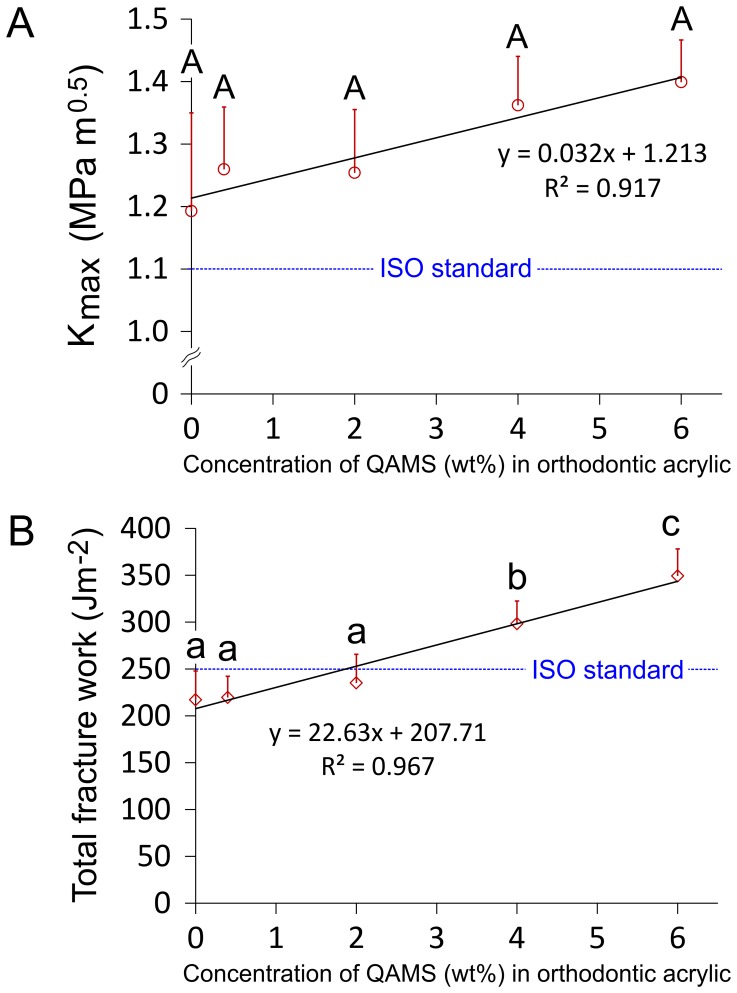
Effects of QAMS concentration on the toughness properties of the orthodontic acrylic resins (N = 10). **A**. Maximum stress intensity factor (K_max_). **B**. Work of Fracture (W_f_). For each parameter, the ISO requirement is included for comparison (dotted line). Linear regression models provide an excellent fit for the relation between K_max_ and QAMS concentration, and the relation between total fracture work and QAMS concentration. For K_max_, groups with the same upper case letter designators are not statistically significant (P>0.05). For total fracture work, groups with the same lower case letter designators are not statistically significant (P>0.05). Values represent means and standard deviations.

The effects of QAMS incorporation on fracture toughness properties of orthodontic acrylic resins are shown in Figure 7. The maximum stress intensity factors (K_max_) for the acrylic resins (Figure 7A) all exceeded the ISO requirement for orthodontic base polymer. No significant difference could be identified among the five groups (P = 0.084). Nevertheless, a highly significant, positive correlation was found between these two parameters (Pearson correlation coefficient  = 0.955; P<0.003). The total fracture work (W_f_) for the acrylic resins are shown in Figure 7B. Acrylic resins containing 6 wt% QAMS had significantly higher W_f_ than those containing 4 wt% QAMS, which, in turn had significantly higher W_f_ than those containing 0–2 wt% QAMS. The QAMS-free acrylic resin failed to meet the ISO requirement for the W_f_ of orthodontic base polymers (250 J/m^2^ for 8 out of 10 specimens). This requirement was met only when more than 2 wt% QAMS was incorporated in the orthodontic acrylic resin. There was a highly significant, positive correlation between W_f_ and QAMS concentration (Pearson correlation coefficient 0.984; P = 0.0004).

**Figure 8:**
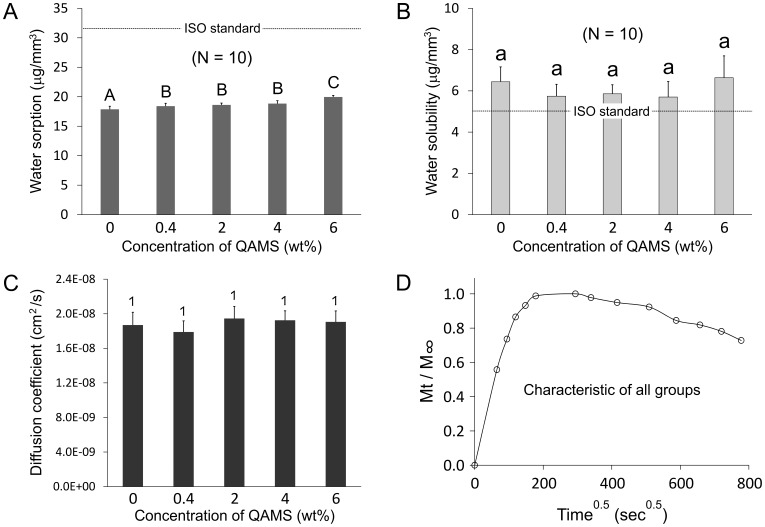
Effects of QAMS concentration on A. Water sorption; B. Water solubility; C. Sorption diffusion coefficients of QAMS-free and QAMS-containing orthodontic acrylic resins (N = 10). Values represent means and standard deviations. ISO requirements for water sorption and water solubility are included for comparison (dotted lines). For each parameter, groups designated with different categories of descriptors (upper case letters for water sorption, lower case letters for water solubility, numerals for diffusion coefficients), are not statistically significant (P>0.05). **D**. A water sorption isotherm representative of all the five groups.

Gravimetric analysis of the water sorption and water solubility characteristics of the 5 groups of orthodontic acrylic, and the variation in the water sorption diffusion coefficients with QAMS concentrations are shown in Figures 8A, 8B, and 8C, respectively. Figure 8D depicts an isotherm that is characteristic of the sorption and solubility profiles among all the 5 groups of resins. Acrylic resins containing 6 wt% QAMS had significantly higher water sorption values than those containing 0.4–2 wt% QAMS, which, in turn, had significantly higher values than the QAMS-free resin (all P<0.05). Nevertheless, these values were much lower than the ISO requirement for water sorption for orthodontic base polymers (32 µg/mm^3^). Although no significant difference was detected for water solubility among the 5 acrylic resin groups, none of the groups was able to meet the ISO requirement for water solubility (5 µg/mm^3^). There was no significant difference in the water diffusion coefficient among the 5 acrylic resin groups.

### Cytotoxicity

For the MTT assay, succinic dehydrogenase activity of the MPDC-23 cells were significantly lower for acrylic resins containing 4 wt% and 6 wt% QAMS before methanol extraction (P<0.05). No difference in succinic dehydrogenase activity could be observed among the QAMS-containing acrylic (0–6 wt%) after methanol extraction. For flow cytometry, the total percentage of apoptotic and necrotic cells was larger for all groups before methanol extraction, compared to the results after methanol extraction. These values were similar to the Teflon negative control, but were much lower than the positive control that was designed to leach a highly toxic material. Taken together, the results indicate that QAMS-containing acrylic resins are generally as biocompatible as QAMS-free acrylic resins, and that cytotoxicity is attributed more to the leached MMA component from polymerized PMMA than to the leached quaternary ammonium species. Details of the results of the two cytotoxicity assays are presented in Figure S4 and Figure S5.

## Discussion

The data obtained from this study support the hypotheses that (1) QAMS-containing orthodontic acrylic resins exhibit antibacterial and antifungal properties; and (2) incorporating QAMS into orthodontic acrylic resins results in improved fracture toughness properties without adversely affecting other physical and mechanical properties.

Microbial biofilms have been associated with many infections and diseases in humans. Sessile microorganisms in the biofilm frequently display properties that are dramatically distinct from their planktonic counterparts [30]. Research has shown that biofilms are at least 500 times more resistant to antibacterial agents than planktonic cells. Various mechanisms have been proposed for such resistance [31], [32], including the inability of antimicrobial chemicals to penetrate biofilms, binding of antimicrobials to the exopolysaccharide matrix of a biofilm [33], and decreases in growth rate of mature biofilm cells, due to nutrition limitation and others [34]. Therefore novel strategies are needed to overcome antimicrobial resistance of the biofilm microorganisms. Immobilizing antimicrobial agents in dental acrylic resin that kills bacteria on contact is a viable alternative. The quaternary ammonium function group in QAMS (SiQAC) exhibits antimicrobial activity against a broad range of microorganisms [35]. Previous studies have shown that SiQAC was successfully used as antimicrobial coatings for fabrics and medical devices [36]–[38]. bisGMA resin containing quaternary ammonium salt with methacrylate group [39], has also exhibited sustained antibacterial effect on *S. mutans* after water-aging for 180 days [40].

Adhesion of microorganisms to a material surface is the initial step in the formation of biofilms [41]. Biofilm formation can be described as a two-phase process, including an initial, instantaneous and reversible physical phase (phase one), followed by a time-dependent and irreversible molecular and cellular phase (phase two) [42]. Many factors are considered to influence bacterial adhesion to material surfaces [43]. It should be noted that the salivary pellicle has an impact on both bacterial [44] and fungal [45] attachment to teeth and prostheses in the oral cavity. In addition, deposition of salivary protein on the material surface might decrease the contact-killing efficiency of an antimicrobial agent. To simulate the oral environment, all microbial biofilms in this study were formed on salivary pellicle-coated acrylic surfaces. In the oral cavity, the acquired pellicle is a biologic layer produced by selective adsorption of salivary proteins on tooth surfaces. This layer acts as the substratum for attachment of oral bacteria including *S. mutans* and *actinomyces* species, in the presence of sucrose. Interactions between these bacteria and the proteins in the acquired pellicle form a very complex process that has been intensively studied in recent years. In addition, surface characteristics such as hydrophobicity and charge density may influence bacterial adhesion and proliferation [46], [47]. While immobilization of QAMS within the PMMA polymer matrix did not inhibit adhesion of *S. mutans* and *A. naselundii*, acrylic resins containing QAMS demonstrated effective contact-killing of the respective biofilms in a dose-dependent manner.

Similar but not the same as bacteria, *C. albicans* biofilm formation is characterized by initial adherence of yeast cells in the first two hours, followed by yeast-hyphal transition, proliferation and maturation within 48 hours [48]. During the adhesion phase, the yeast cells bind to a surface coated with a glycoproteinaceous conditioning film [49]. In the present study, contrasting to *S. mutans* and *A. naeslundii*, surface adhesion of *C. albicans* was inhibited by QAMS, in a dose-dependent manner. This observation may have been attributed to the increased surface hydrophobicity with increasing concentration of QAMS. Minagi *et al*. [50] have reported enhanced adhesion of *C. albicans* to acrylic resin with surface free energy approximating that of *C. albicans*. Such attachment mechanism is based on hydrophobic interaction between *C. albicans* and the acrylic resin.

The results derived from fluorescein staining indicate that quaternary ammonium groups were retained on the orthodontic acrylic surfaces after water aging. This is comparable to a recent study when similar concentrations of a quaternary ammonium methacrylate were incorporated into resins used for preparing tooth-colored filling materials [25]. Although leaching of quaternary ammonium species was also detected by the bromophenol blue assay, this only occurred when a high concentration of QAMS (6 wt%) was incorporated in the acrylic. Moreover, recent studies have shown that quaternary ammonium silanes (SIQAC) that leached out of substrates had no residual antimicrobial activity [51], due to association phenomena in aqueous media, such as micellization, formation of ion-pairs and small aggregates [52]. These deactivation processes are significantly enhanced in the presence of NaCl [51]. Although these leachate deactivation phenomena may not be immediately apparent within the biovolume of a biofilm, the fact that QAMS are present on the acrylic surface after the leaching experiments indicate that contact-killing will be the predominant killing mechanism in QAMS-containing acrylic, particularly after the initially-formed biofilms are removed by cleaning and tooth brushing.

In addition to antimicrobial properties, it is important for the orthodontic acrylic resin copolymerized with QAMS to have comparable mechanical and physical properties, as removable appliances prepared from acrylic resin fracture easily, particularly along the midline, due to repeated masticatory loads and frequent withdrawal and replacement of those appliances. Reinforcing acrylic resins with high modulus glass or polyethylene fibers is a possible strategy for improving their mechanical properties [53]. Nevertheless, such reinforcement may be compromised, due to failure to achieve true adhesion between the fibers and the methacrylate resin matrix [54]. After copolymerizing with methyl methacrylate, the QAMS improved the fracture toughness of the acrylic resin and its ability to absorb energy (total fracture work), while maintaining flexural strength and moduli comparable to that of the QAMS-free, control resin. Incorporating as little as 0.6 wt% of a different type of antibacterial quaternary ammonium methacrylate, methacryloyloxyundecylpyridinium bromide (MUPB), into an acrylic resin was found to reduce the flexural strength significantly [55]. In contrast, acrylic resin containing less than 2 wt% QAMS exhibited a flexural strength comparable with the QAMS-free, control resin. Another major advantage of the use of QAMS over MUPB is that MUPB is only antibacterial, and is not antifungal [56].

Although addition of the QAMS up to 4–6 wt% yields a relatively high percentage of dead bacteria within the body of the biofilms, there is concern that unreacted QAMS in the polymerized resin may leach out during aging in water. From the ATR-FTIR results, the presence of methacrylate C = C peak at 1636 cm^−1^ for polymerized acrylic containing 4 and 6 wt% QAMS suggests reduced overall conversion in these resins. However, these C = C peaks may also be representative of methacrylate bonds of unreacted silane functional ends of the QAMS, where at least one of the multiple linkages has reacted with the PMMA polymer chain, thus creating a covalently bound, non-leachable, pendent methacrylate group. While the bromophenol blue assay provided an indirect indication of the presence of quaternary ammonium species in the leachate, the exact nature of the quaternary ammonium species (e.g. hydrolysis product derived from QAMS) should be further investigated using gas chromatography (GC) or high performance liquid chromatography (HPLC) in future work.

Hydrophobicity of the acrylic resin was increased as QAMS concentration was elevated. A previous study also reported decreased wettability of silica surfaces coated by quaternary ammonium silane [57]. The simultaneous increases in hydrophobicity and water sorption of the QAMS-containing acrylic resins appear to be contradictory. However, one must realize that the so-called increase in water sorption in the ISO standards is based only on mass measurements. Copolymerization of QAMS with PMMA could have produced co-polymer network or an interpenetrating polymer network with smaller pore sizes. This formation could have restricted the outward diffusion of heavier, unpolymerized components (compared with water), while the inward diffusion of water is hampered by the increase in hydrophobicity of the QAMS-containing resin. In addition, all 5 groups of acrylic resins failed to meet the ISO requirement for water solubility. This result may be attributed to the dissolution of the phthalate plasticizer from the resin system.

A non-leaching antimicrobial agent should have a long-term effect. Thus, further studies are needed to test the retention of antimicrobial activity in dental materials over time. Although the results of the present study indicate definitive improvement in toughness of the QAMS-containing acrylic resin, it should be noted that fracture toughness evaluation is usually performed using low displacement rates (*ca*. 1 mm/min). In contrast, impact occurs using much higher displacement rates, and is a very important phenomenon in governing the life of a structure under function. For example, impact fracture can take place when an orthodontic acrylic appliance is accidentally dropped to the floor, or when a patient generates a sudden impact force, as in chewing ice. Hence, future work should be performed on the impact resistance of orthodontic acrylic resins containing QAMS [58].

## Supporting Information

Figure S1 **Schematic of the rotating disk reactor employed for growth of biofilms.** **A**. Nutrient bottle for culture broth and sucrose supplement. **B**. Perfusion pump. **C**. Reactor vessel. **D**. Waste bottle. **E**. Specimen holder. **F**. Recesses for holding orthodontic acrylic resin disks. **G**. Photograph of the biofilm reactor placed inside an anaerobic chamber for growing *S. mutans* and *A. naeslundii* biofilms under anaerobic conditions.
(TIF)

Figure S2 **^29^Si CP-MAS NMR of completely hydrolyzed and condensed QAMS.** The NMR spectrum revealed two broad resonance peaks at −68.37 ppm that are assigned to the silicon in the RSi(OSi)_3_ species (T^3^ bonding), and at −58.41 that is assigned to the silicon RSi(OH)(OSi)_2_ moiety (T^2^ bonding). These broad peaks are indicative of the heterogeneous nature of the condensate, with molecules containing -Si-O-Si-, -Si-O-Si-O-Si, and -Si-O-Si-O-Si-O-Si siloxane bridges, and possibly others within the network formation. The presence of T^3^ and T^2^ functionalities confirms the existence of the covalent linkage between the organic alkoxy groups and the silicate backbone. No Q-series (siloxane, single silanol and germinal silanols) could be identified from the spectrum, indicating that the final condensed product is an organically modified silicate (ORMOSIL).
(TIF)

Figure S3 **Representative ATR-FTIR spectra of components of the QAMS and resin-based materials used and made in the study.** **Panel A**. The totally hydrolyzed and condensed QAMS. **Panel B**. The orthodontic resin (in the uncured and cured states). **Panel C**. The polymerized QAMS-containing PMMA material.
(TIF)

Figure S4 **Cytotoxicity studies on acrylic disks containing different concentrations of QAMS before and after methanol (MeOH) extraction (to remove remnant methyl methacrylate monomers within the processed acrylic).** Teflon was used as negative control and Intermediate Restorative Material (IRM), a zinc oxide eugenol-based material, was used as the positive control. A. MTT assay of cell metabolism (mitochrondrial succinic dehydrogenase activity). For comparison of activities before MeOH extraction, groups denoted by different asterisk symbols are significantly different from those without symbols (p<0.05). For comparison of activities after MeOH extraction, the group indicated by “†” is significantly different from groups without symbols (p<0.05). For before and after MeOH extraction comparisons, adjacent columns from each group that are labeled with a horizontal bar are significantly different (p<0.05). **B**. Flow cytometry results, comparing the total percent of apoptotic ad necrotic MDPC-23 cells after exposure to the orthodontic acrylic specimens. Although these two tests examined different aspects of cell viability, the results of the MTT assay and flow cytometry are complementray, and indicate that QAMS-containing acrylic resins are generally as biocompatible as QAMS-free acrylic resins, and that cytotoxicity is attributed more to the leached MMA component from polymerized PMMA than to the leached quaternary ammonium species.
(TIF)

Figure S5 **Two-dimensional dot plots of MDPC-23 cells after exposure to IRM and orthodontic acrylic specimens.** Cells were stained with FITC-Annexin V (green fluorescence) and ethidium homodimer III (red fluorescence), to determine the percentage distribution of viable (lower left quadrant), early apoptotic (lower right quadrant), late apoptotic (upper right quadrant) and necrotic cells (upper left quadrant). **A**. Before methanol extraction. **B**. After methanol extraction.
(TIF)

Text S1 **Experimental details: Cytotoxicity of QAMS-containing orthodontic acrylic resins.**
(DOCX)

Text S2 **Results Interpretation: ATR-FTIR of components of the QAMS and resin-based materials used and made in the study.**
(DOCX)

## References

[1] King G. Access to Orthodontic Services in the US. American Association of Orthodontics website. Available: http://www.aaomembers.org/mtgs/upload/King-Access-to-Orthodontic-Care-The-Problem-and-Some-Solutions.pdf. Accessed 2012 May 1.

[2] Bjerklin K, Gärskog B, Rönnerman A (1983) Proximal caries increment in connection with orthodontic treatment with removable appliances. Br J Orthod 10: 21–24. 6573195 10.1179/bjo.10.1.21

[3] Tamura K, Nakano K, Miyake S, Takada A, Ooshima T (2005) Clinical and microbiological evaluations of acute periodontitis in areas of teeth applied with orthodontic bands. Ped Dent J 15: 212–218.

[4] Hibino K, Wong RW, Hägg U, Samaranayake LP (2009) The effects of orthodontic appliances on Candida in the human mouth. Int J Paediatr Dent 19: 301–308. 19486368 10.1111/j.1365-263X.2009.00988.x

[5] Batoni G, Pardini M, Giannotti A, Ota F, Giuca MR, et al (2001) Effect of removable orthodontic appliances on oral colo-nization by mutans streptococci in children. Eur J Oral Sci 109: 388–392. 11767275 10.1034/j.1600-0722.2001.00089.x

[6] Arendorf TM, Addy M (1985) Candidal carriage and plaque distribution before, during and after removable orthodontic appliance therapy. J Clin Periodontol 12: 360–368. 3859496 10.1111/j.1600-051x.1985.tb00926.x

[7] Morgan TD, Wilson M (2000) Anti-adhesive and antibacterial properties of proprietary denture cleanser. J Appl Microbiol 89: 617–623. 11054165 10.1046/j.1365-2672.2000.01158.x

[8] Lessa FCR, Enoki C, Ito IY, Matsumoto MA, Nelson-Filho P (2007) In-vivo evaluation of the bacterial contamination and disinfection of acrylic baseplates of removable orthodontic appliances. Am J Orthod Dentofacial Orthop 131: 705.e11–7. 10.1016/j.ajodo.2006.09.042 17561044

[9] Vento-Zahra E, De Wever B, Decelis S, Mallia K, Camilleri S (2011) Randomized, double-blind, placebo-controlled trial to test the efficacy of nitradine tablets in maxillary removable orthodontic appliance patients. Quintessence Int 42: 37–43. 21206932

[10] Sodagar A, Kassaee MZ, Akhavan A, Javadi N, Arab S, et al (2012) Effect of silver nano particles on flexural strength of acrylic resins. J Prosthodont Res 56: 120–124. 21835724 10.1016/j.jpor.2011.06.002

[11] Kassaee MZ, Akhavan A, Sheikh N, Sodagar A (2008) Antibacterial effects of a new dental acrylic resin containing silver nanoparticles. J Appl Polym Sci 110: 1699–1703.

[12] Wady AF, Machado AL, Zucolotto V, Zamperini CA, Berni E, et al (2012) Evaluation of Candida albicans adhesion and biofilm formation on a denture base acrylic resin containing silver nanoparticles. J Appl Microbiol 112: 1163–1172. 22452416 10.1111/j.1365-2672.2012.05293.x

[13] Monteiro DR, Gorup LF, Takamiya AS, de Camargo ER, Filho AC, et al (2012) Silver distribution and release from an antimicrobial denture base resin containing silver colloidal nanoparticles. J Prosthodont 21: 7–15. 22050139 10.1111/j.1532-849X.2011.00772.x

[14] Oei JD, Zhao WW, Chu L, Desilva MN, Ghimire A, et al (2012) Antimicrobial acrylic materials with in situ generated silver nanoparticles. J Biomed Mater Res B Appl Biomater 110B: 409–415. 10.1002/jbm.b.31963 22102276

[15] Shinonaga Y, Arita K (2012) Antibacterial effect of acrylic dental devices after surface modification by fluorine and silver dual-ion implantation. Acta Biomater 8: 1388–1393. 21971415 10.1016/j.actbio.2011.09.017

[16] Rantala LI, Lastumäki TM, Peltomäki T, Vallittu PK (2003) Fatigue resistance of removable orthodontic appliance reinforced with glass fibre weave. J Oral Rehabil 30: 501–506. 12752930 10.1046/j.1365-2842.2003.01108.x

[17] Gong SQ, Niu LN, Kemp LK, Yiu CKY, Ryou H, et al (2012) Quaternary ammonium silane-functionalized, methacrylate resin composition with antimicrobial activities and self-repair potential. Acta Biomater. doi:10.1016/j.actbio.2012.05.031. 10.1016/j.actbio.2012.05.031 PMC3580770 22659173

[18] Ahlström B, Thompson RA, Edebo L (1999) The effect of hydrocarbon chain length, pH, and temperature on the binding and bactericidal effect of amphiphilic betaine esters on Salmonella typhimurium. APMIS 107: 318–324. 10223305 10.1111/j.1699-0463.1999.tb01560.x

[19] Tiller JC, Liao CJ, Lewis K, Klibanov AM (2001) Designing surfaces that kill bacteria on contact. Proc Natl Acad Sci USA 98: 5981–5985. 11353851 10.1073/pnas.111143098 PMC33409

[20] Owen MJ (1980) The surface activity of silicones: A short review. Ind Eng Chem Prod Res Dev 19: 97–103.

[21] International Organization for Standardization. ISO 20795–2: Dentistry – base polymers. Part 2: Orthodontic base polymers. 1^st^ ed. Geneva: The Organization.

[22] Hentzer M, Teitzel GM, Balzer GJ, Heydorn A, Molin S, et al (2001) Alginate overproduction affects Pseudomonas aeruginosa biofilm structure and function. J Bacteriol 183: 5395–5401. 11514525 10.1128/JB.183.18.5395-5401.2001 PMC95424

[23] Chávez de Paz LE (2009) Image analysis software based on color segmentation for characterization of viability and physiological activity of biofilms. Appl Environ Microbiol 75: 1734–1739. 19139239 10.1128/AEM.02000-08 PMC2655470

[24] Ozer RR, Cary Hill W, Rogers ME, Evans M (2010) Development of colormetric analytical methods to monitor quaternary amine grafted surfaces. J Appl Polym Sci 118: 2397–2407.

[25] Antonucci JM, Zeiger DN, Tang K, Lin-Gibson S, Fowler BO, et al (2012) Synthesis and characterization of dimethacrylates containing quaternary ammonium functionalities for dental applications. Dent Mater 28: 219–228. 22035983 10.1016/j.dental.2011.10.004 PMC3259208

[26] Yamamoto K (1995) Sensitive determination of quaternary ammonium salts by extraction spectrophotometry of ion associates with bromophenol blue anion and coextraction. Analytica Chimica Acta 302: 75–79.

[27] Yiu CK, King NM, Carrilho MR, Sauro S, Rueggeberg FA, et al (2006) Effect of resin hydrophilicity and temperature on water sorption of dental adhesive resins. Biomaterials 27: 1695–1703. 16226310 10.1016/j.biomaterials.2005.09.037

[28] Vahdat N, Sullivan VD (2000) Estimation of permeation rate of chemical through elastometric materials. J Appl Polym Sci 79: 1265–1272.

[29] Leggat PA, Kedjarune U (2003) Toxicity of methyl methacrylate in dentistry. Int Dent J 53: 126–131. 12873108 10.1111/j.1875-595x.2003.tb00736.x

[30] Costerton JW, Lewandowski Z, Caldwell DE, Korber DR, Lappin-Scott HM (1995) Microbial biofilms. Annu Rev Microbiol 49: 711–745. 8561477 10.1146/annurev.mi.49.100195.003431

[31] Lewis K (2001) Riddle of biofilm resistance. Antimicrob Agents Chemother 45: 999–1007. 11257008 10.1128/AAC.45.4.999-1007.2001 PMC90417

[32] Mahmoud TF, O’Toole GA (2001) Mechanisms of biofilm resistance to antimicrobial agents. Trends Microbiol 9: 34–39. 11166241 10.1016/s0966-842x(00)01913-2

[33] Ishida H, Ishida Y, Kurosaka Y, Otani T, Sato K, et al (1998) In vitro and in vivo activities of levofloxacin against biofilm-producing *Pseudomonas aeruginosa* . Antimicrob Agents Chemother 42: 1641–1645. 9660997 10.1128/aac.42.7.1641 PMC105659

[34] Costerton JW, Stewart PS, Greenberg EP (1999) Bacterial biofilms: a common cause of persistent infections. Science 284: 1318–1322. 10334980 10.1126/science.284.5418.1318

[35] Isquith AJ, Abbott EA, Walters PA (1972) Surface-bonded antimicrobial activity of an organosilicon quaternary ammonium chloride. Appl Microbiol 24: 859–863. 4650597 10.1128/am.24.6.859-863.1972 PMC380687

[36] Gottenbos B, van der Mei HC, Klatter F, Nieuwenhuis P, Busscher HJ (2002) In vitro and in vivo antimicrobial activity of covalently coupled quaternary ammonium silane coatings on silicone rubber. Biomaterials 23: 1417–1423. 11829437 10.1016/s0142-9612(01)00263-0

[37] Oosterhof JJ, Buijssen KJ, Busscher HJ, van der Laan BF, van der Mei HC (2006) Effects of quaternary ammonium silane coatings on mixed fungal and bacterial biofilms on tracheoesophageal shunt prostheses. Appl Environ Microbiol 72: 3673–3677. 16672516 10.1128/AEM.72.5.3673-3677.2006 PMC1472352

[38] Monticello RA, White WC (2009) Inhibition of foundation colonization of biofilm by surface modification with organofunctional silanes. In: Paulson DS, ed. Applied Biomedical Microbiology: A Biofilms Approach. 45–58.

[39] Cheng L, Weir MD, Xu HH, Antonucci JM, Kraigsley AM, et al (2012) Antibacterial amorphous calcium phosphate nanocomposites with a quaternary ammonium dimethacrylate and silver nanoparticles. Dent Mater 28: 561–572. 22305716 10.1016/j.dental.2012.01.005 PMC3322309

[40] Cheng L, Weir MD, Zhang K, Xu SM, Chen Q, et al (2012) Antibacterial nanocomposite with calcium phosphate and quaternary ammonium. J Dent Res 91: 460–466. 22403412 10.1177/0022034512440579 PMC3327730

[41] Hori K, Matsumoto S (2010) Bacterial adhesion: From mechanism to control. Biochem Eng J 48: 424–434.

[42] An YH, Friedman RJ (1997) Laboratory methods for studies of bacterial adhesion. J Microbiol Meth 30: 141–152.

[43] Katsikogianni M, Missirlis YF (2004) Concise review of mechanisms of bacterial adhesion to biomaterials and of techniques used in estimating bacteria-material interactions Eur Cell Mater. 8: 37–57. 10.22203/ecm.v008a05 15593018

[44] Pratt-Terpstra IH, Weerkamp AH, Busscher HJ (1989) The effects of pellicle formation on streptococcal adhesion to human enamel and artificial substrata with various surface free-energies. J Dent Res 68: 463–467. 2921388 10.1177/00220345890680030501

[45] Jin Y, Samaranayake LP, Samaranayake Y, Yip HK (2004) Biofilm formation of Candida albicans is variably affected by saliva and dietary sugars. Arch Oral Biol 49: 789–798. 15308423 10.1016/j.archoralbio.2004.04.011

[46] Harkes G, Feijen J, Dankert J (1991) Adhesion of Escherichia coli on to a series of poly(methacrylates) differing in charge and hydrophobicity. Biomaterials 12: 853–860. 1764557 10.1016/0142-9612(91)90074-k

[47] Speranza G, Gottardi G, Pederzolli C, Lunelli L, Canteri R, et al (2004) Role of chemical interactions in bacterial adhesion to polymer surfaces. Biomaterials 25: 2029–2037. 14741617 10.1016/j.biomaterials.2003.08.061

[48] Chandra J, Kuhn DM, Mukherjee PK, Hoyer LL, McCormick T, et al (2001) Biofilm formation by the fungal pathogen Candida albicans: development, architecture, and drug resistance. J Bacteriol 183: 5385–5394. 11514524 10.1128/JB.183.18.5385-5394.2001 PMC95423

[49] Cannon RD, Chaffin WL (1999) Oral colonization by Candida albicans. Crit Rev Oral Biol Med 10: 359–383. 10759414 10.1177/10454411990100030701

[50] Minagi S, Miyake Y, Inagaki K, Tsuru H, Suginaka H (1985) Hydrophobic interaction in Candida albicans and Candida tropicalis adherence to various denture base resin materials. Infect Immun 47: 11–14. 3880719 10.1128/iai.47.1.11-14.1985 PMC261449

[51] Song L, Baney RH (2011) Antibacterial evaluation of cotton textile treated by trialkoxysilane compounds with antimicrobial moiety. Text Res J 81: 504–511.

[52] Kopecky F (1996) Micellization and other associations of amphiphilic antimicrobial quaternary ammonium salts in aqueous solutions. Pharmazie 51: 135–144. 8900863

[53] Kanie T, Fujii K, Arikawa H, Inoue K (2000) Flexural properties and impact strength of denture base polymer reinforced with woven glass fibers. Dent Mater 16: 150–158. 11203537 10.1016/s0109-5641(99)00097-4

[54] Grave AM, Chandler HD, Wolfaardt JF (1985) Denture base acrylic reinforced with high modulus fibre. Dent Mater 1: 185–187. 3867574 10.1016/s0109-5641(85)80015-4

[55] Regis RR, Zanini AP, Della Vecchia MP, Silva-Lovato CH, Oliveira Paranhos HF, et al (2011) Physical properties of an acrylic resin after incorporation of an antimicrobial monomer. J Prosthodont 20: 372–379. 21627706 10.1111/j.1532-849X.2011.00719.x

[56] Regis RR, Della Vecchia MP, Pizzolitto AC, Compagnoni MA, Souza PPC, et al (2012) Antimicrobial Properties and Cytotoxicity of an Antimicrobial Monomer for Application in Prosthodontics. J Prosthodont 21: 283–290. 22339776 10.1111/j.1532-849X.2011.00815.x

[57] Song J, Kong H, Jang J (2011) Bacterial adhesion inhibition of the quaternary ammonium functionalized silica nanoparticles. Colloids Surf B Biointerfaces 82: 651–656. 21115282 10.1016/j.colsurfb.2010.10.027

[58] Zappini G, Kammann A, Wachter W (2003) Comparison of fracture tests of denture base materials. J Prosthet Dent 90: 578–585. 14668759 10.1016/j.prosdent.2003.09.008

