# Supplementary figures and images for: An ORMOSIL-Containing Orthodontic Acrylic Resin with Concomitant Improvements in Antimicrobial and Fracture Toughness Properties
Source: PLoS One. 2012 Aug 1;7(8):e42355. doi: 10.1371/journal.pone.0042355 (PMC3411672; doi:10.1371/journal.pone.0042355)

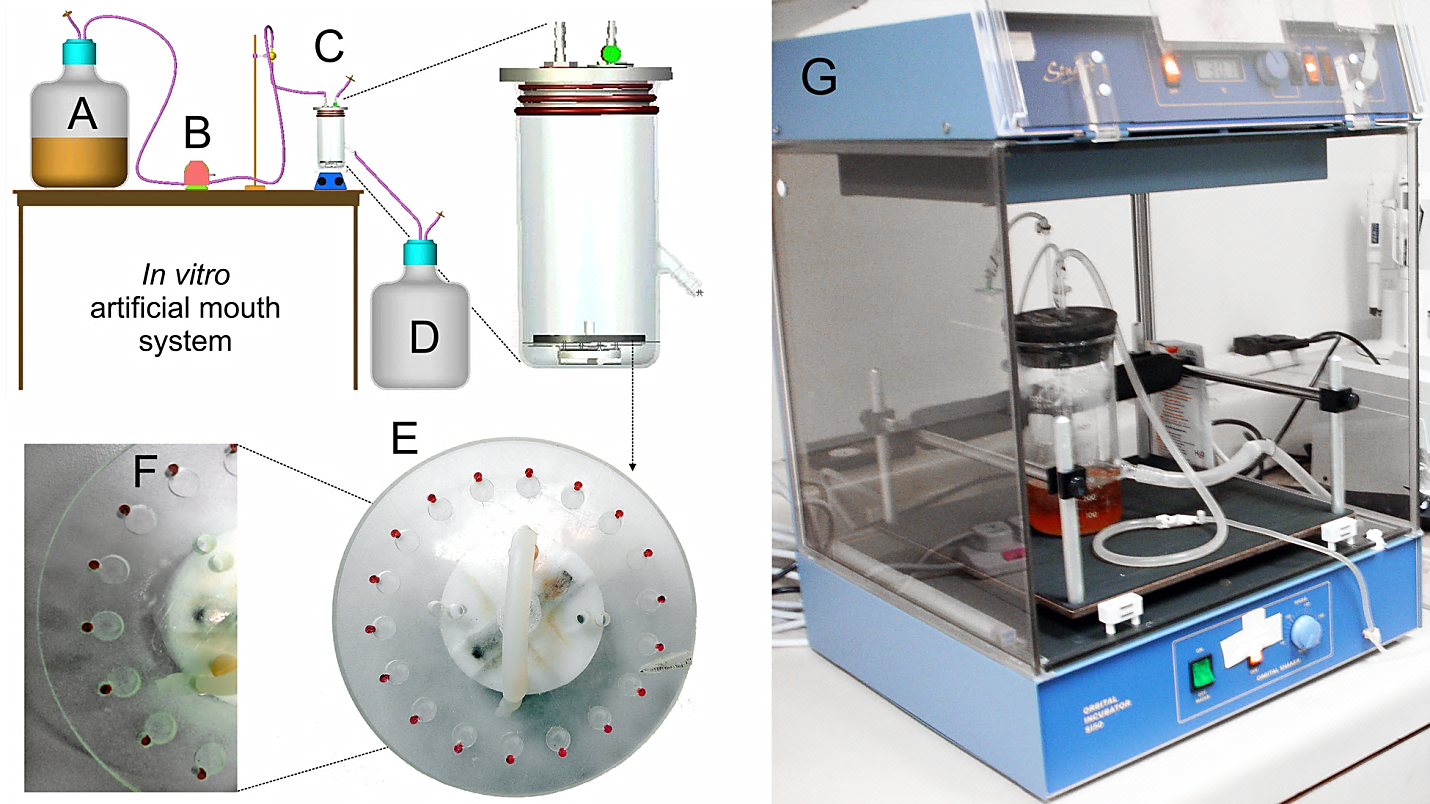

Supplement: Figure S1 — Schematic of the rotating disk reactor employed for growth of biofilms. A. Nutrient bottle for culture broth and sucrose supplement. B. Perfusion pump. C. Reactor vessel. D. Waste bottle. E. Specimen holder. F. Recesses for holding orthodontic acrylic resin disks. G. Photograph of the biofilm reactor placed inside an anaerobic chamber for growing S. mutans and A. naeslundii biofilms under anaerobic conditions. (TIF) [file pone.0042355.s001.tif]

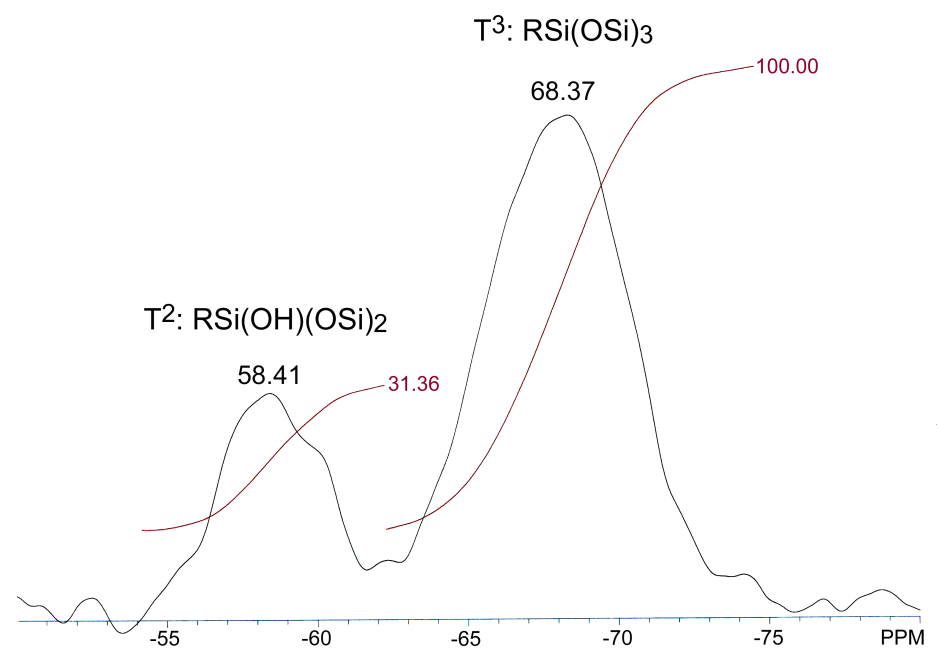

Supplement: Figure S2 — 29Si CP-MAS NMR of completely hydrolyzed and condensed QAMS. The NMR spectrum revealed two broad resonance peaks at −68.37 ppm that are assigned to the silicon in the RSi(OSi)3 species (T3 bonding), and at −58.41 that is assigned to the silicon RSi(OH)(OSi)2 moiety (T2 bonding). These broad peaks are indicative of the heterogeneous nature of the condensate, with molecules containing -Si-O-Si-, -Si-O-Si-O-Si, and -Si-O-Si-O-Si-O-Si siloxane bridges, and possibly others within the network formation. The presence of T3 and T2 functionalities confirms the existence of the covalent linkage between the organic alkoxy groups and the silicate backbone. No Q-series (siloxane, single silanol and germinal silanols) could be identified from the spectrum, indicating that the final condensed product is an organically modified silicate (ORMOSIL). (TIF) [file pone.0042355.s002.tif]

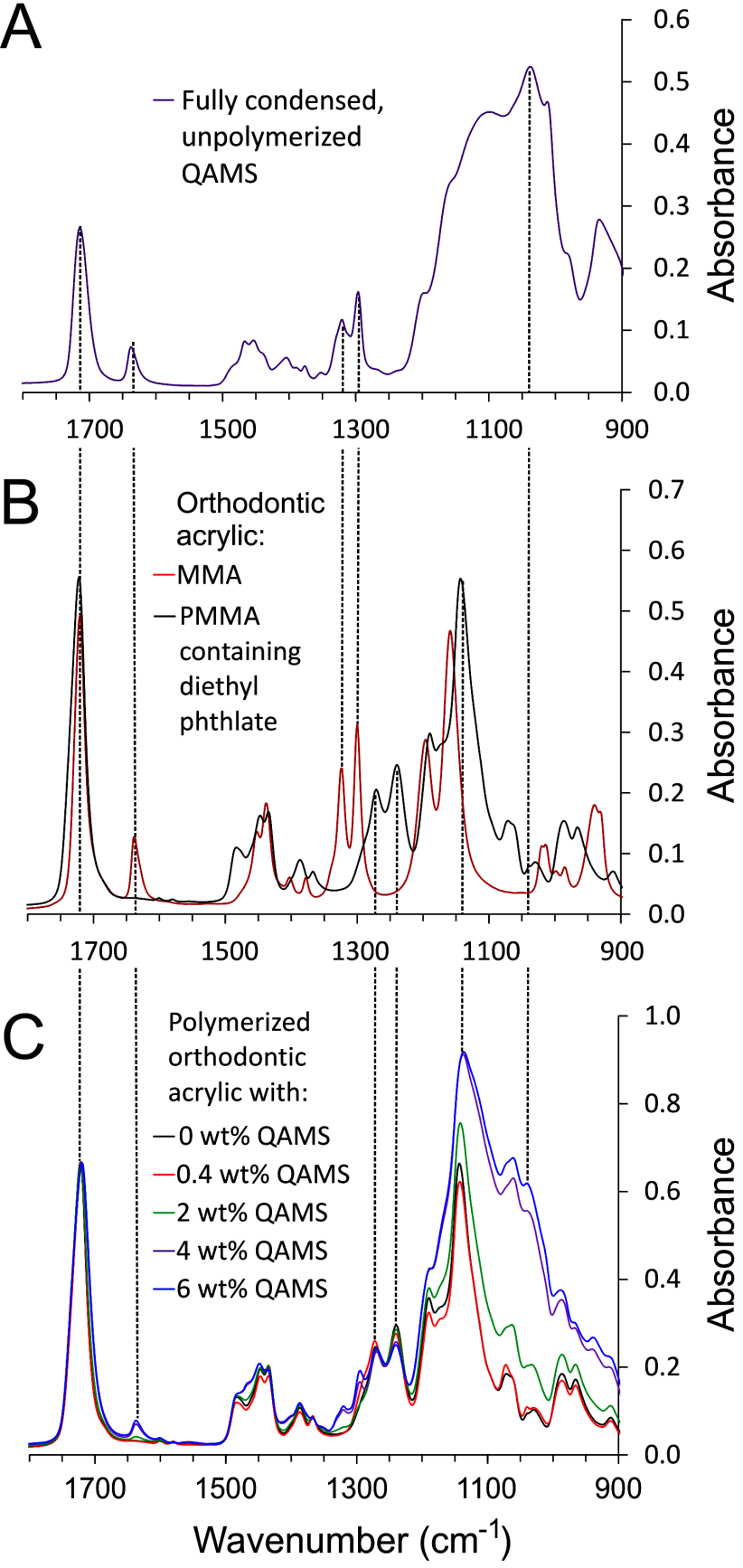

Supplement: Figure S3 — Representative ATR-FTIR spectra of components of the QAMS and resin-based materials used and made in the study. Panel A. The totally hydrolyzed and condensed QAMS. Panel B. The orthodontic resin (in the uncured and cured states). Panel C. The polymerized QAMS-containing PMMA material. (TIF) [file pone.0042355.s003.tif]

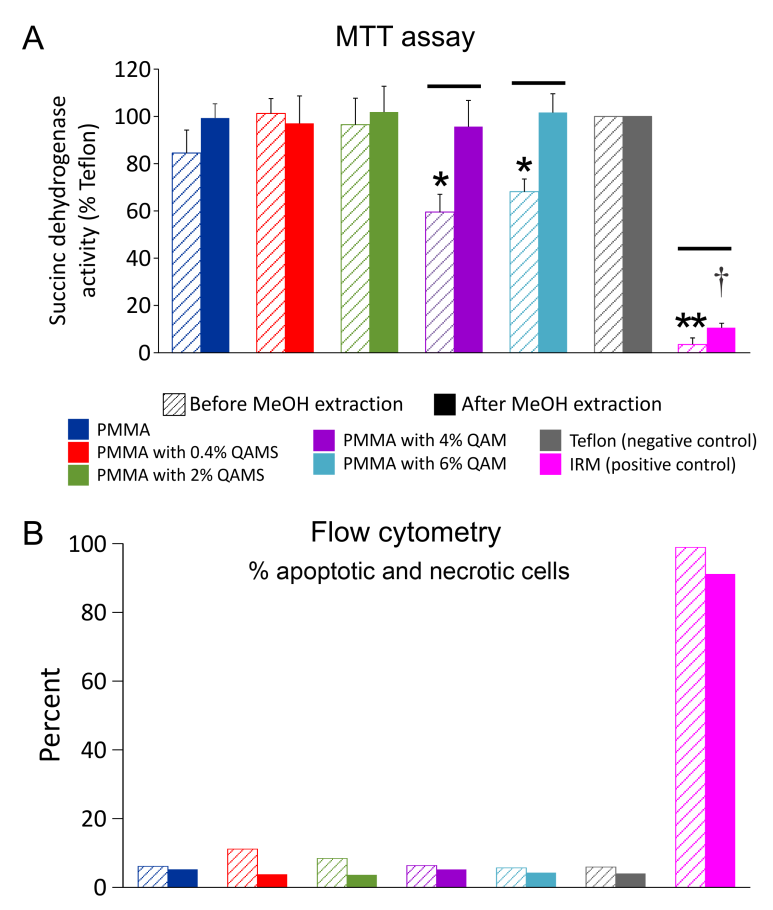

Supplement: Figure S4 — Cytotoxicity studies on acrylic disks containing different concentrations of QAMS before and after methanol (MeOH) extraction (to remove remnant methyl methacrylate monomers within the processed acrylic). Teflon was used as negative control and Intermediate Restorative Material (IRM), a zinc oxide eugenol-based material, was used as the positive control. A. MTT assay of cell metabolism (mitochrondrial succinic dehydrogenase activity). For comparison of activities before MeOH extraction, groups denoted by different asterisk symbols are significantly different from those without symbols (p<0.05). For comparison of activities after MeOH extraction, the group indicated by “†” is significantly different from groups without symbols (p<0.05). For before and after MeOH extraction comparisons, adjacent columns from each group that are labeled with a horizontal bar are significantly different (p<0.05). B. Flow cytometry results, comparing the total percent of apoptotic ad necrotic MDPC-23 cells after exposure to the orthodontic acrylic specimens. Although these two tests examined different aspects of cell viability, the results of the MTT assay and flow cytometry are complementray, and indicate that QAMS-containing acrylic resins are generally as biocompatible as QAMS-free acrylic resins, and that cytotoxicity is attributed more to the leached MMA component from polymerized PMMA than to the leached quaternary ammonium species. (TIF) [file pone.0042355.s004.tif]

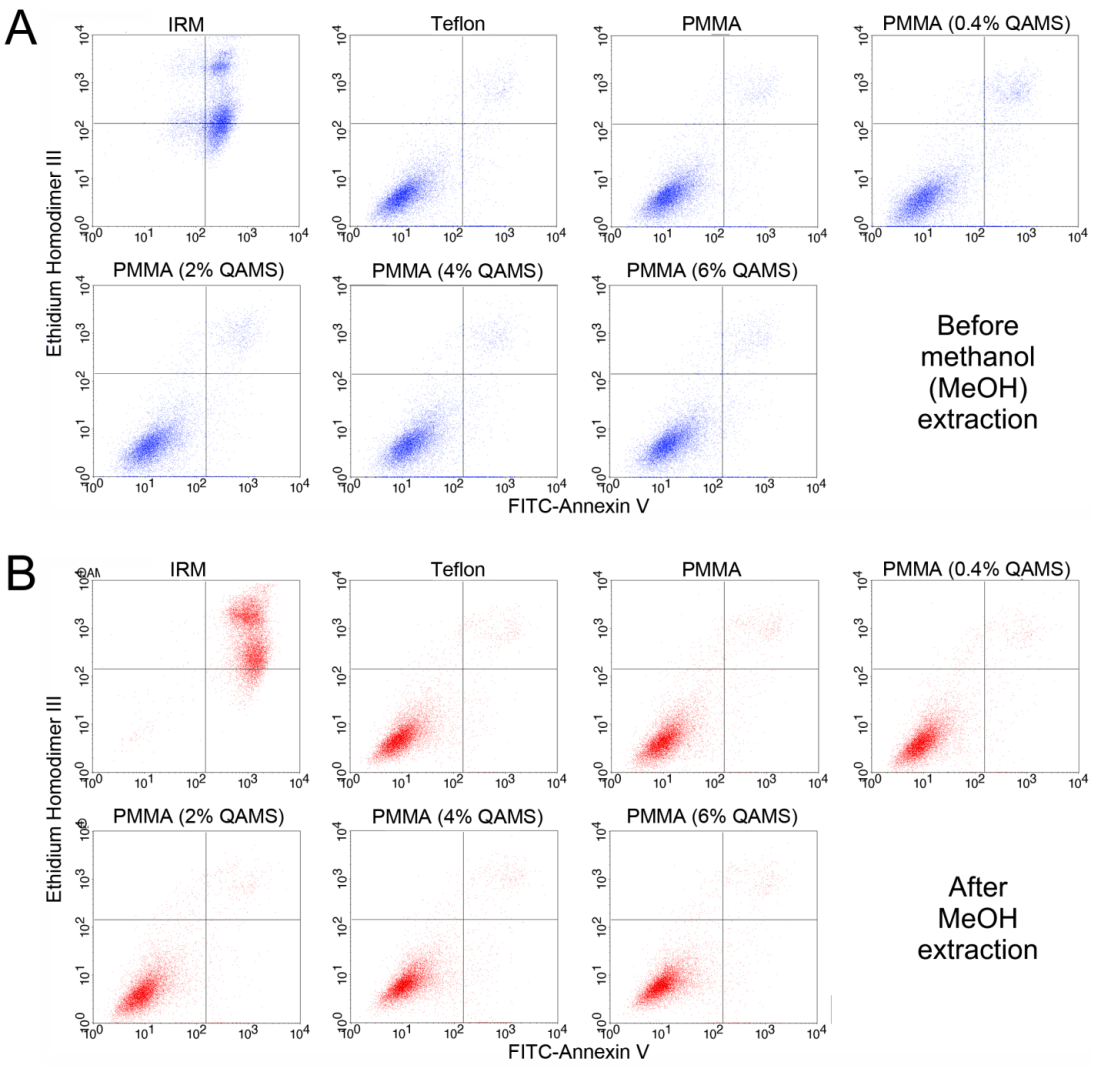

Supplement: Figure S5 — Two-dimensional dot plots of MDPC-23 cells after exposure to IRM and orthodontic acrylic specimens. Cells were stained with FITC-Annexin V (green fluorescence) and ethidium homodimer III (red fluorescence), to determine the percentage distribution of viable (lower left quadrant), early apoptotic (lower right quadrant), late apoptotic (upper right quadrant) and necrotic cells (upper left quadrant). A. Before methanol extraction. B. After methanol extraction. (TIF) [file pone.0042355.s005.tif]
