# Supplementary material for: An ORMOSIL-Containing Orthodontic Acrylic Resin with Concomitant Improvements in Antimicrobial and Fracture Toughness Properties
Source: PLoS One. 2012 Aug 1;7(8):e42355. doi: 10.1371/journal.pone.0042355 (PMC3411672; doi:10.1371/journal.pone.0042355)
Supplement: Text S1 — Experimental details: Cytotoxicity of QAMS-containing orthodontic acrylic resins. (DOCX) [file pone.0042355.s006.docx]

**Text S1 Experimental details: Cytotoxicity of QAMS-containing orthodontic acrylic resins**

***Cell Culture***

The cytotoxicity of QAMS-containing acrylic resins was investigated using a murine dental papilla-derived odontoblast-like cell line (MDPC-23). The cells were plated in a 24-well format at a density of 5,000 cells/cm2, in 0.5 mL of the growth medium, and incubated at 37 °C in a humidified 5% CO_2_ atmosphere for 24 hours until fully established. The growth medium consisted of Dulbecco's Modified Eagle's Medium (DMEM, Lonza, Wakersville, MD, USA) and 10% fetal bovine serum (Gibco, Invitrogen Corp., Carlsbad, CA, USA), supplemented with 2 mmol/L L-glutamine and 100 U/mL penicillin-streptomycin.

The materials tested included the QAMS-containing acrylic resins (0, 0.4, 2, 4 and 6 wt% QAMS), Teflon (negative control), and Intermediate Restorative Material (IRM), a zinc oxide eugenol-based material that releases cytotoxic eugenol (positive control). The materials were prepared in the form of 5-mm diameter, 3-mm thick disks, and were sterilized with ultraviolet light for 4 hours prior to testing. As remnant MMA within the polymerized PMMA acrylic is cytotoxic, experiments were performed before and after the processed QAMS-containing acrylic specimens and the control specimens were subjected to methanol extraction of the residual MMA. Methanol extraction was performed by immersing the specimen disks in methanol for 24 h. The disks were removed and air-dried prior to sterilization with ultraviolet light.

The sterilized disks (N = 12) were placed individually in transwell inserts with 3 μm pore size (BD Falcon, Franklin Lakes, NJ) to prevent direct contact of cells by the specimen. After the inserts were placed over the plated cells, an additional 2 mL of growth medium was added to each well to ensure that the level of the culture medium was above the sides of the transwell insert. The disks were exposed to the plated cells for 3 days, without further change in culture medium, prior to testing.

***MTT Assay***

Cell viability was evaluated by incubating 3-(4,5-dimethylthiazol-2-yl)-2,5-diphenyltetrazolium bromide (MTT) with MDPC-23 cells that have been exposed to the specimen disks placed over the transwell inserts, as described above. The MTT assay was used to evalute the ability of the cells to provide energy for cell function and growth. This is because cellular damage by toxic biomaterials reduces the ability of the cells to metabolize tetrazolium salts via mitochondrial dehydrogenases involved in the citric acid cycle and the electron transport chain. Accordingly, the cells were incubated in MTT-succinate solution for 60 min and fixed with Tris-formalin. The purple MTT-formazan produced in the cells, as a result of mitochrondrial succinc dehydrogenase activity, was dissolved *in-situ* using DMSO-NaOH and the optical density was measured using a microplate reader at 562 nm. The optical density of blank DMSO-NaOH was subtracted from all wells. The formazan content of each well was computed as a percentage of the mean of the Teflon controls, which was taken to represent 100% biocompatibility.

Data derived from the experimental and control groups, before and after methanol extraction, were subjected to logarithmic transformation to comply with the normality and homoscedasticity assumptions of parametric statistical analysis methods. The transformed data was analyzed with a two-factor repeated measures ANOVA to determine the effects of methanol extraction and material composition on succinic dehydrogenase activity. When the omnibus test indicated statistically significant results, Tukey-Kramer post-hoc analyses were used for comparing the QAMS-acrylic formulations to the control formulations, with statistical significance preset at α = 0.05.

***Flow Cytometry***

Flow cytometry was used to examine the effect of the scaffolds on cell death-induced plasma membrane permeability to fluorescent dyes and DNA stains. The MDPC-23 cells were plated at the same density as previously described and exposed to the aforementioned described experimental and control formulations (both before and after methanol extraction) for 3 days. The cells were then washed twice with phosphate-buffered saline and detached from the culture wells with 0.25% trypsin. The harvested cells were centrifuged to discard the supernatant and re-suspended at 1x10^4^ cells/mL, in 1X binding buffer included in the Apoptosis and Necrosis Quantification Kit (Biotium, Inc., Hayward, CA, USA). The cells were stained with fluorescein isothiocyanate-Annexin V (FITC-AnV; λ_abs_/λ_em_= 492/514 nm; green fluorescence) and ethidium homodimer-III (Etd; λ_abs_/λ_em_ = 528/617 nm; red fluorescence), and incubated for 15 min in the dark. The stained MDPC-23 cells were subjected to fluorescence-activated cell sorting (FACS) using a FACSCalibur flow cytometer (BD Biosciences, San Jose, CA, USA), to determine the percentage distribution of viable (AnV/Etd negative), early apoptotic (AnV positive, Etd negative), late apoptotic (secondary necrosis; AnV/Etd positive) and necrotic cells (AnV negative, Etd positive). As pooled cells derived from multiple wells were required, cell sorting was only performed once. Thus, no statistical analysis was performed to analyze the results.
