# Supplementary material for: An ORMOSIL-Containing Orthodontic Acrylic Resin with Concomitant Improvements in Antimicrobial and Fracture Toughness Properties
Source: PLoS One. 2012 Aug 1;7(8):e42355. doi: 10.1371/journal.pone.0042355 (PMC3411672; doi:10.1371/journal.pone.0042355)
Supplement: Text S2 — Results Interpretation: ATR-FTIR of components of the QAMS and resin-based materials used and made in the study. (DOCX) [file pone.0042355.s007.docx]

**Text S1 Results Interpretation: ATR-FTIR of components of the QAMS and resin-based materials used and made in the study.**

The carbonyl peak (1718 cm^-1^) can be identified in all spectra, as well as characteristic peaks associated with the C=C bond itself. In the QAMS product (Panel A), C=C peaks are present as unreacted methacrylate groups of the attached silane groups: at 1636 cm^-1^ and the two peaks at 1321 and 1298 cm^-1^. There is a broad absorption from 1200 cm^-1^ to 960 cm^-1^, peaking at 1040 cm^-1^. This non-descript region represents the various Si-O-Si bond formations, most notable being that of the 1040 cm^-1^ peak. The many absorption peaks between 1100 and 1200 cm^-1^ representing organic functional groups present in the uncured C=C state add to the general overlapping of spectra and the absence of isolated, individual absorption data.

The spectra in Panel B shows the effect of formation of aliphatic, polymeric bonds as well as decrease in the effect of the C=C group after polymerization. Using the second, third, and forth vertical dotted lines from the left of the panels, it can be seen that both the QAMS and the PMMA resins contain characteristic peaks identifying the presence of C=C. Note the general absence of the Si-O-Si peak in Panel B, seen at 1040 cm^-1^ in Panel A.

Panel C presents the spectral overlay of orthodontic acrylic resins made using various weight percentage values of the QAMS component, with spectra normalized to the height of the carbonyl peak at 1718 cm^-1^. Note that formulations containing 4 and 6 wt% QAMS also indicate the appearance of the methacrylate C=C in their polymerized profiles: at 1636, 1321, and 1298 cm^-1^, suggesting that addition of the QAMS component to this extent reduces overall conversion. However, these C=C peaks may also be representative of methacrylate bonds of unreacted silane functional ends of the QAMS, where at least one of the multiple linkages has reacted with the PMMA polymer chain, thus creating a covalently bound, unleachable, pendent methacrylate group. Such distinction between the bound and freely leachable components is not possible using this methodology. The absence of these absorbance values suggests high utilization of that group, inferring elevated levels of monomer conversion among the 0%, 0.4% and 2% QAMS formulations. Also note the high peak intensities associated with functional groups forming the polymer network: the two vertical lines appearing at 1266 and 1235 cm^-1^. In addition, spectra contain varying levels of absorbance values at 1140 cm^-1^. This band may be associated with symmetric stretching of the vinyl ester group, indicating lower conversion with increasing QAMS content. The presence of the Si-O-Si bond associated with the QAMS component is present as a group of absorptions (1000 to 1100 cm^-1^), and represents a multitude of possible siloxane combinations: general Si-O, disiloxanes, linear, cyclic trimer, and cyclic tetramer. The extent to which each of these species contributes to the absorption profiles in Panels A and C cannot be determined, because of the concomitant presence of absorptions near the same frequency range associated with the presence of functional groups present in the organic components of the organically modified silicate (ORMOSIL). That said, the peak occurring in Panel A near 1040 cm^-1^ may be associated with the Si-O-Si bond, because none of the organic components have a specific absorption at this location. Thus, looking closely at Panel C, a small shoulder can be identified that indicates the presence of Si-O-Si groups within the polymerized material (dotted vertical, right-most line).
